# Photothermal-Responsive Phase Transition of Proteoliposomes for Heat Shock Protein Sequestering against Cancer Thermoresistance

**DOI:** 10.34133/research.1231

**Published:** 2026-03-31

**Authors:** Kai Cheng, Yongxu Han, Fang Zhang, Biquan Li, Bin Zeng, Yuan-Di Zhao, Jiang Xia

**Affiliations:** ^1^ Department of Chemistry, The Chinese University of Hong Kong, Hong Kong SAR, China.; ^2^ MOE Key Laboratory for Biomedical Photonics - Hubei Bioinformatics & Molecular Imaging Key Laboratory, Department of Biomedical Engineering, College of Life Science and Technology, Huazhong University of Science and Technology, Wuhan 430074, Hubei, China.; ^3^ AoE Centre for Plant Vacuole Biology and Biotechnology, The Chinese University of Hong Kong, Hong Kong SAR, China.

## Abstract

Photothermal therapy (PTT) has garnered considerable attention for its noninvasive and localized treatment advantages. However, in response to PTT-induced hyperthermia, cancer cells increase the expression level of heat shock proteins (HSPs) and activate thermoresistance to shield themselves from heat-induced damage, thereby diminishing the efficacy of PTT. To overcome thermoresistance, here we have developed an on-demand responsive proteoliposome (PL) system. This system consists of PLs formed by a phospholipid conjugate of an elastin-like polypeptide (ELP) with vanadium oxide nanozymes (VO_x_ NZs) incorporated in the lumen, referred to as VO_x_@ELP-PL. Upon photoirradiation, the enclosed VO_x_ NZs generate a photothermal effect, inducing hyperthermia and enhancing HSP expression in cancer cells. Concurrently, as the temperature surpasses a critical threshold, ELP-PL undergoes liquid–liquid phase separation (LLPS) in situ, transitioning from a liposome state to ELP coacervate droplets. In the hyperthermic cancer cells, ELP coacervate droplets sequester and insulate the up-regulated HSPs, disrupting the thermoprotective response of thermoresistant cancer cells. Moreover, VO_x_@ELP-PL combines peroxidase-catalyzed generation of toxic hydroxyl radicals with coacervate droplet-mediated sequestration of HSPs, leading to potentiated immunogenic cell death both in vitro and in vivo. In a mouse model of colon cancer, intravenously injected VO_x_@ELP-PL showed marked tumor enrichment and resulted in highly effective cancer treatment. Altogether, this system presents a novel strategy to counteract thermoresistance by sequestering HSPs via LLPS of ELP-PL, thereby augmenting the effectiveness of PTT in cancer therapy.

## Introduction

Photothermal therapy (PTT) uses light-generated heat to induce the death of cancer cells. It offers advantages such as noninvasiveness, deep tissue penetration, and precise spatiotemporal control [1–3]. PTT typically uses photothermal conversion agents (PCAs), such as near-infrared (NIR) light-responsive nanomaterials [4–6], to induce hyperthermia in cancer cells (temperatures exceeding 42 °C), effectively destroying cancer cells and enabling tumor ablation or even complete tumor eradication. Additionally, PTT can reprogram the immunosuppressive “cold” tumor microenvironment and enhance the recovery of innate immune function [7,8].

Among all the PCAs, vanadium oxide nanozymes (VO_x_ NZs) are particularly interesting. Vanadium compounds have been proposed for the treatment of diabetes, cancer, and infectious diseases [9–11]. Being one of the 14 essential trace elements for the human body, vanadium has a mix of valence versatility, affordability, and nontoxicity, making it an appealing choice for developing nanozymes with various biomedical applications [12,13]. Moreover, VOx efficiently absorbs and converts NIR light into heat with a high photothermal conversion efficiency [14]. For example, we demonstrated that VO_x_ NZs in their +4 valence state can kill 4T1 breast cancer cells through an oxidation reaction, producing highly toxic hydroxyl radicals [15]. Zhang et al. [16] developed biodegradable vanadium-based nanomaterials to enhance PTT at 1,064 nm through ferroptosis and pyroptosis. Vanadium-iron oxide nanoparticles were also developed to destroy hepatoma cells through combining photothermal and chemodynamic therapies [17].

Despite the potential of PTT in cancer treatment, the effectiveness of PCA-mediated cell killing is limited by a self-defense mechanism, where cancer cells in a hyperthermic state activate antiapoptotic and cytoprotective pathways, resulting in thermoresistance, particularly within the “hyperthermia range” (42 to 47 °C) [18–22]. Cancer cells respond to heat stress by overexpressing heat shock proteins (HSPs) as a self-protection strategy; this overexpression increases heat tolerance, alleviates heat-induced stress, and decreases PTT efficiency. HSPs are a family of adenosine triphosphate-dependent chaperone molecules that serve various roles in regulating signal transduction and protect cells from adverse conditions, such as heat [23,24]. In many types of cancer, the expression of HSPs is markedly increased, making HSPs reliable biomarkers for the disease [25]. Moreover, its overexpression is positively correlated with poor prognosis and resistance to multiple therapies. During hyperthermia, tumor cells quickly overexpress HSPs to repair damaged proteins and activate antiapoptotic mechanisms, such as inhibiting caspase-3 activation [26], which reduces the effectiveness of PTT.

Various strategies have been developed to counteract thermoresistance and sensitize cancer cells to heat-induced cytotoxicity. For instance, PTT was combined with other therapeutic modalities, such as photodynamic therapy, chemotherapy, gene therapy, or immunotherapy, to achieve synergistic antitumor effects [27–34]. A viable strategy is to inhibit the production or activity of heat-induced HSPs to counteract the thermoresistance. For example, we reported that cobalt ions can enhance the effect of PTT by locally preventing heat resistance through binding to HSP90 [35]. HSP inhibitors or specific small interfering RNAs (siRNAs) have been utilized to decrease thermoresistance in PTT [36–40]. For example, Liu et al. [36] coadministered an HSP inhibitor triptolide through the in situ release from a temperature and redox-dual-sensitive nanoreservoir. Alternatively, Gu and coworkers developed a photothermal platform consisting of a hollow gold nanoshell core densely loaded with siRNAs against HSP70. When exposed to NIR light irradiation, siRNAs can detach from the gold surface and escape from endosomes to silence HSP70 [19]. However, these methods face limitations. The inhibitory effect of small molecules or siRNA often lags behind the rapid photothermal response, as siRNA must enter the cytosol to take effect. Moreover, each small molecule or siRNA typically targets only one specific HSP. Therefore, a system capable of rapidly and broadly lowering all HSP levels to block thermoresistance is desirable.

On another note, under specific conditions, proteins tend to form coacervate droplets through liquid–liquid phase separation (LLPS) [41–43]. These protein coacervate droplets may enrich other proteins or nucleic acids, forming structurally independent particles known as membraneless organelles, and participate in a wide range of cellular processes [44–46]. In particular, elastin-like polypeptides (ELPs) are polymeric protein sequences composed of repetitive elastin peptides, such as Pro-Gly-Val-Pro-Gly-Val. ELPs display lower critical solution temperature (LCST)-type phase transition behavior, meaning they are soluble at low temperatures and insoluble at high temperatures. The temperature sensitivity of ELP is because the hydrophobic amino acid residues are hidden at low temperatures; as the temperature increases, these hydrophobic regions cluster together, causing protein aggregation and precipitation [47–50]. Appropriately designed ELPs can undergo LLPS and form condensates or coacervate droplets above the LCST [51]. Because of the photothermal controllability of vanadium oxide, we hypothesize that synergizing the photothermal effect of VO_x_ NZs with the thermal-responsive coacervation of ELPs will enable sequential control of phase transition via NIR light.

**Fig. 1.**
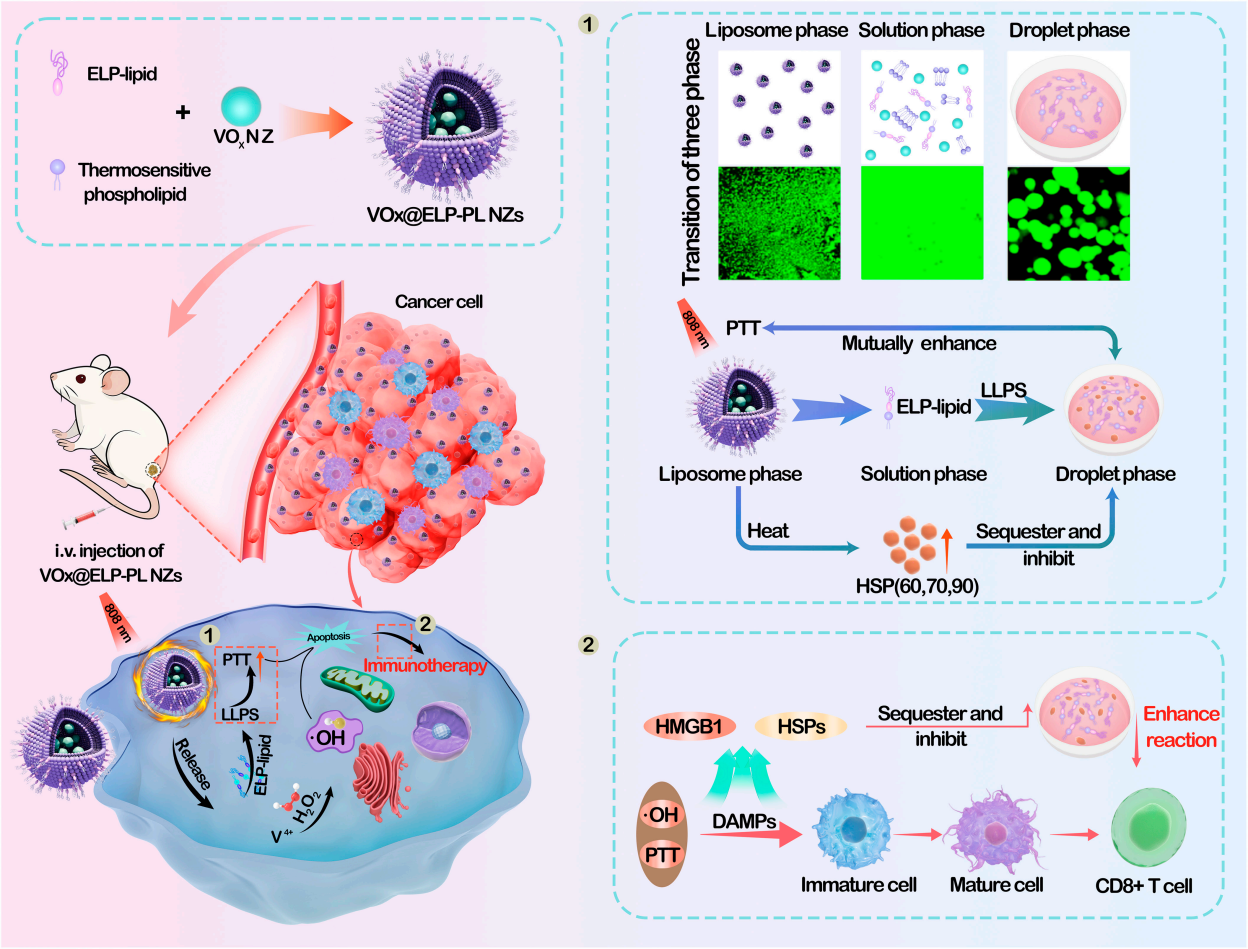
Schematic diagrams showing the construction of VO_x_@ELP-PL and the mechanism of action to induce cancer cell death. Elastin-like polypeptide (ELP)-proteoliposome (PL) encapsulates vanadium oxide nanozymes (VO_x_ NZs) and shields the immediate cytotoxicity of NZs. When enriched at tumor sites, irradiation with near-infrared light (NIR, 808 nm) disrupts the thermosensitive PL, releasing VO_x_ NZs to generate V^5+^ and V^4+^, unleashing cytotoxicity. Simultaneously, the photothermal effect of VO_x_ NZs elevates the temperature above the critical temperature of ELP-PL, promoting the phase transition to give ELP coacervate droplets, which sequester heat shock proteins (HSPs) and inhibit thermoprotection. Photothermal effects combined with damage-associated molecular pattern (DAMP) release enhance CD8^+^ T cell production, thereby achieving efficient treatment of colorectal cancer (CRC) in vitro and in vivo.

In this work, we have designed a thermoresponsive proteoliposome (PL) system formed by an ELP–phospholipid conjugate. The ELP–phospholipid conjugate forms ELP-PL at room temperature and undergoes a phase transition to form ELP coacervate droplets in the form of liquid droplets through LLPS above the critical phase-transition temperature. Encapsulating the PCA, VO_x_ NZs, within the interior of the ELP-PL creates a photothermal-responsive VO_x_@ELP-PL. When delivered into cancer cells, illumination of VO_x_@ELP-PL with light raises the temperature in tumor tissue above 45 °C. The temperature rise triggers the phase transition of ELP-PL to form ELP coacervate droplets in situ within cells, which enrich and sequester the overexpressed HSPs. “Sequestration” here is defined as the physical compartmentalization of proteins within coacervate droplets, which may lead to “sequestration” or functional inhibition by restricting molecular interactions. Such a combined effect mitigates the stress-sensing and protective roles of HSPs, enhancing the photothermal effect of VO_x_ NZs. Moreover, degradation of VO_x_ NZs releases V^5+^ and V^4+^ ions (V^5+^ is further reduced to V^4+^), and the Fenton-like reaction of V^4+^ produces hydroxyl radicals, which synergize with damage-associated molecular patterns (DAMPs) to induce immunogenic cell death (ICD) (Fig. 1). Taken together, the interplay of photothermal VO_x_ NZs and liposome-to-coacervate transition sensitizes PTT against cancer thermoresistance.

## Results

### Design, construction, and characterization of VO_x_@ELP-PL

Temperature-sensitive PL is designed based on the thermoresponsive phase transition properties of ELPs: at temperatures below the LCST, ELPs exist in the solution state; increasing the temperature causes the condensation of ELPs to form phase-separated coacervate droplets via LLPS [48]. We hypothesize that conjugating ELP with a phospholipid will give an ELP–phospholipid (ELP–lipid) conjugate that can undergo thermoresponsive phase transition from the PL state at low temperatures to coacervate droplets at higher temperatures. Based on this hypothesis, we synthesized an ELP–lipid conjugate and formed PL, which we refer to as ELP-PL.

**Fig. 2.**
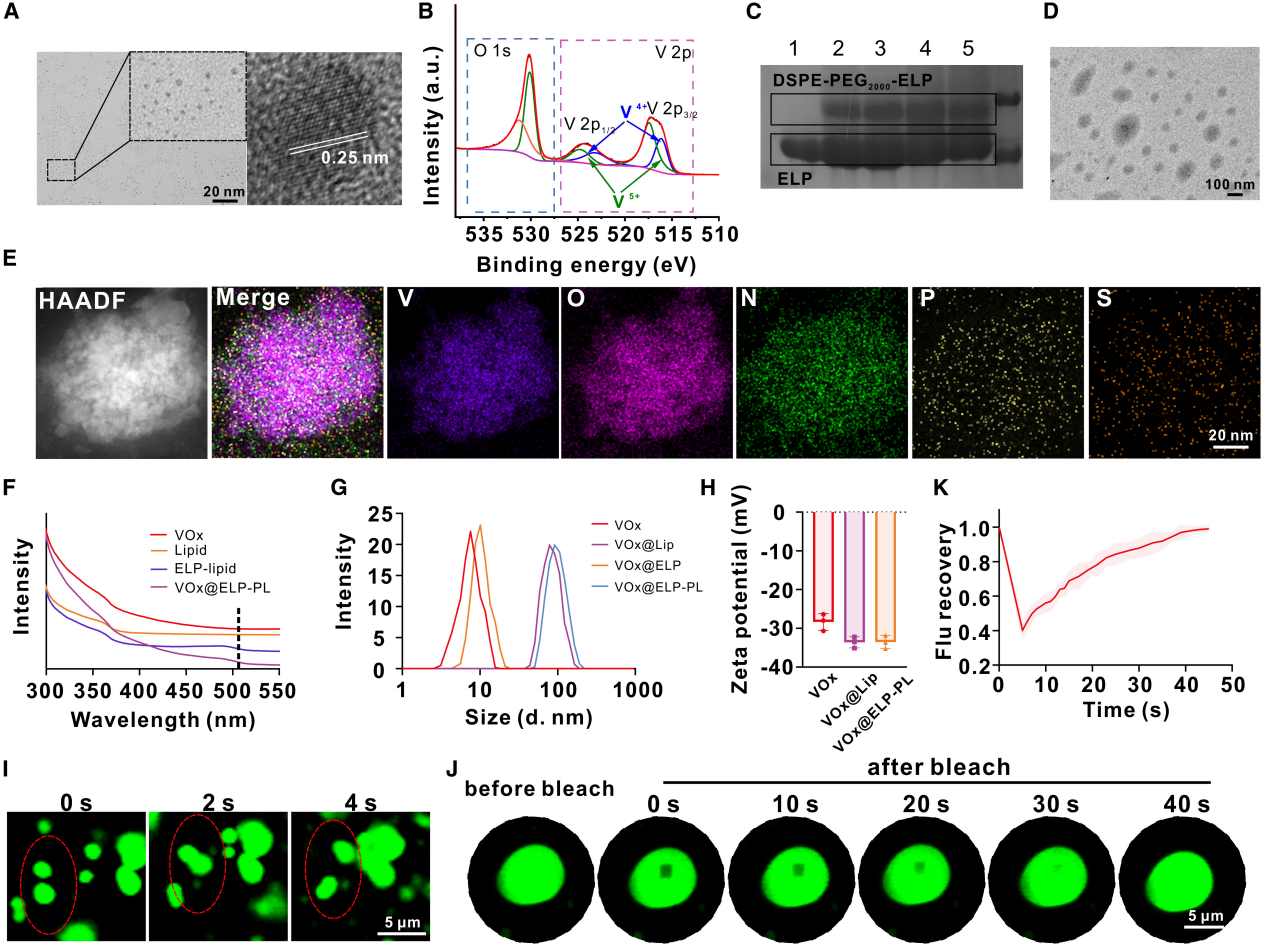
Characterization of VO_x_@ELP-PL. (A) Transmission electron microscopy (TEM) images and lattice magnification of VO_x_ NZs. (B) X-ray photoelectron spectroscopy (XPS) valence analysis of VO_x_. (C) The sodium dodecyl sulfate–polyacrylamide gel electrophoresis (SDS-PAGE) after the coupling of DSPE-PEG_2000_-NHS and ELP. Briefly, the molar ratio of ELP to DSPE-PEG_2000_-NHS was set to 1:10, and lanes 2 to 5 represent different reaction pH values (7.2, 8.3, 8.5, and 9.0). (D and E) TEM and element mapping of VO_x_@ELP-PL. (F) Ultraviolet–visible (UV–Vis) absorption spectrum of VO_x_, Lip, ELP–lipid, and VO_x_@ELP-PL. (G) Hydrated particle size of VO_x_, VO_x_@Lip, VO_x_@ELP, and VO_x_@ELP-PL. (H) Zeta potential of VO_x_, VO_x_@Lip, and VO_x_@ELP-PL. (I) ELP–lipid spontaneously fuses with each other at 45 °C. Data are presented as mean ± standard deviation (*n* = 6 independent samples). (J) The photobleached region of the ELP–lipid coacervate droplets spontaneously recovers the fluorescence after 40 s. The excitation and emission wavelengths were set at 488 and 525 nm, respectively. (K) Quantification of the fluorescence recovery after photo-bleaching (FRAP) analysis.

Next, we synthesized VO_x_ NZs as PCA using redox etching. Transmission electron microscopy (TEM) results showed that VO_x_ NZs have granular shapes with sizes of about 5 nm and a lattice distance of 0.25 nm (Fig. 2A). Valence state analysis based on x-ray photoelectron spectroscopy showed that VO_x_ had 2 valence states, pentavalent V^5+^ and tetravalent V^4+^, with proportions of 64.95% and 35.05%, respectively. Characteristic peaks were found at 516.13 eV for V 2p 3/2 and 523.07 eV for V 2p1/2, showing the presence of V^4+^, and 517.42 and 524.74 eV for V^5+^ (Fig. 2B). On another note, sodium dodecyl sulfate–polyacrylamide gel electrophoresis (SDS-PAGE) analysis indicated successful synthesis of ELP (Fig. S1) and ELP–lipid conjugates after the reaction of ELP with DSPE-PEG_2000_-NHS (Fig. 2C). ELP–lipid conjugates form ELP-PL, which showed diameters of about 100 nm under TEM (Fig. 2D).

Next, we encapsulated VO_x_ NZs within ELP-PL to give VO_x_@ELP-PL. Element analysis and energy dispersive spectrometer (EDS) spectrum analysis (Fig. 2E and Fig. S2) demonstrated the presence of V, O, N, P, and S signals in the composite particles of VO_x_@ELP-PL (V and O from VO_x_ NZs, and N, P, and S are from ELP–lipid conjugates). From the absorption spectrum, the VO_x_@ELP-PL possessed a obvious absorption peak around 480 nm (Fig. 2F). Dynamic light scattering results showed that after simple ELP modification, the particle size of VO_x_ did not change markedly, whereas formulation with ELP-PL led to a marked increase in particle size (Fig. 2G). They also confirmed a relatively uniform dispersion of the particles (Fig. S3). According to the zeta potential results, VO_x_@Lip and VO_x_@ELP-PL possessed similar surface charges (Fig. 2H). Differential scanning calorimetry was performed to analyze the liposomes and ELP-PL samples (Fig. S4). The results showed that ELP modification reduced the enthalpy change (Δ*H*) associated with the lipid phase transition, indicating successful integration of the thermosensitive ELP component and its influence on the bilayer organization. Furthermore, encapsulation of VO_x_ within ELP-PL led to a further pronounced decrease in Δ*H* compared to ELP modification alone. This additional reduction strongly suggests that the VO_x_ nanoparticles are confined within the aqueous lumen of the liposomes, where they introduce distinct perturbations in the lipid bilayer architecture, thereby providing direct evidence of successful VO_x_ encapsulation. Under elevated temperatures (>45 °C), ELP–lipid microdroplets in the aqueous solution could fuse into larger droplets (Fig. 2J), showing mobility and liquid-like features. When a portion of the ELP–lipid droplet was photo-bleached by laser light, the fluorescence gradually recovered to its original strength within 40 s (Fig. 2K and I). All the above results indicated that (a) ELP–lipid conjugate forms PL that can encapsulate VO_x_ NZs, and (b) ELP–lipid conjugate can undergo LLPS to form ELP coacervate droplets.

### Photothermal properties of VO_x_@ELP-PL

**Fig. 3.**
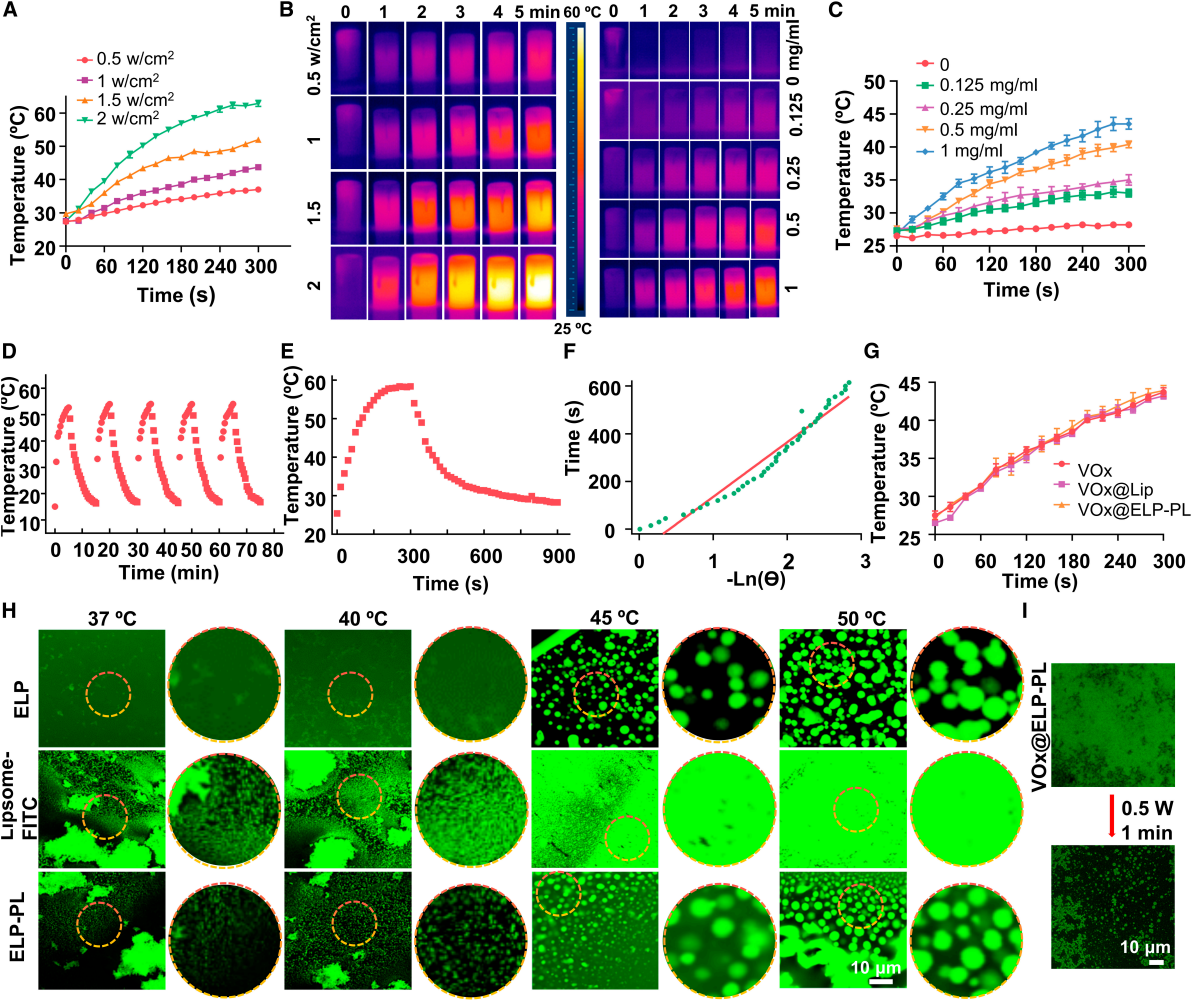
Photothermal-responsive phase transition of VO_x_@ELP-PL. (A to C) Temperature changes of VO_x_ NZs (1 mg/ml) under different laser irradiation (0.5, 1, 1.5, and 2 W/cm^2^) and different concentrations of VO_x_ under the same laser irradiation (1 W/cm^2^), and corresponding infrared thermal imaging. The data are presented as mean ± standard deviation (*n* = 3 independent samples). (D) Temperature changes of VO_x_ NZs (1 mg/ml) under repeated cycles of laser irradiation (2 W/cm^2^). (E and F) Rise–fall temperature of VO_x_, and calculation of the *τ* value chart. Here, the laser power was set at 0.8 W, and the absorption value *A*_808_ of the VO_x_ NZs solution at 808 nm was 0.86, calculated using the formula $\eta =\frac{\mathrm{hs}\left(T_{\mathrm{Max}}-T_{\text{Surr}}\right)-Q_{\mathrm{Dis}}}{I\left(1-10^{-A_{808}}\right)}$. (G) Temperature changes of VO_x_, VO_x_@Lip, and VO_x_@ELP-PL under 1 W/cm^2^ laser irradiation. Data are presented as mean ± standard deviation (*n* = 3 independent samples). (H) Confocal imaging of ELP, Liposome-FITC, and ELP-PL at different temperatures (37, 40, 45, and 50 °C). The excitation and emission wavelengths were set at 488 and 525 nm, respectively. (I) Confocal microscope images of VO_x_@ELP-PL before and after laser (808 nm) irradiation at 0.5 W (spot area: 5 mm × 7 mm).

The photothermal property of VO_x_ NZs was first examined. Under the same VO_x_ concentration (1 mg/ml), higher power of laser irradiation on VO_x_ NZs induced higher temperature increase, proving the photothermal property of the NZs (Fig. 3A and B). Also, under the same laser irradiation power (1 W/cm^2^), higher VO_x_ NZ concentration corresponded to a higher temperature rise (Fig. 3B and C). For example, at 1 mg/ml of VO_x_ NZs and a laser power density of 1.5 W/cm^2^, the temperature of the solution rose 20 °C within 5 min. Repeated laser irradiation could induce multiple cycles of temperature rise, indicating that VO_x_ NZs have a good photothermal stability (Fig. 3D). Since *T*_max_ was 58.4 °C and *T*_sur_ was 28.3 °C, the photothermal conversion efficiency of VO_x_ NZ solution was calculated to be 23.99% [34] (Fig. 3E and F). Encapsulation in the liposome did not change the photothermal properties of VO_x_ NZs (Fig. 3G). These data showed that VO_x_ NZs displayed robust photothermal properties, which rapidly and reversibly increased the solution temperature by responding to photoirradiation.

Next, we evaluated the phase separation behavior of ELP, liposome, and ELP-PL at different temperatures (37 to 50 °C). After heating for 1 min, the turbidity at 600 nm was tested, and it was found that the turbidity of ELP-PL was lower at 37 and 40 °C, but higher at 45 and 50 °C, which was also significantly different from that at 37 °C (Fig. S5). The fluorescence microscopy images showed that at 37 and 40 °C, ELP existed in the solution state, while at 45 and 50 °C, coacervate droplets formed in the solution. DSPE formed liposomes at lower temperatures; when the temperature rose above 45 °C, the liposome structures disappeared. ELP-PL, on the other hand, showed a unique thermal-responsive phase transition property: at 37 and 40 °C, ELP-PL existed as liposomes; at 45 and 50 °C, ELP-PL phase transited to coacervate droplets, mimicking LLPS of ELP protein (Fig. 3H). We also analyzed the phase transition behavior of both ELP and ELP–lipid (Fig. S6). The results showed that when the temperature exceeded 45 °C, the absorbance of both ELP and ELP–lipid at 395 nm began to increase, suggesting a phase transition from the soluble state to the coacervate droplets state. As the temperature continued to rise, the absorbance continued to increase, suggesting gradual stabilization of the liquid–liquid phase-separated state. The phase transition behavior of ELP-PL was also found in VO_x_@ELP-PL. Photo-irradiation with an 808-nm laser caused the gradual change of VO_x_@ELP-PL from the liposome state to liquid coacervate droplets (Fig. 3I), indicating that the photothermal effect of VO_x_ NZs converted photo energy to heat, which disrupted the liposome structure and induced phase transition of ELP-PL to coacervate droplets. Altogether, these data show that the photothermal response of VO_x_ NZs encapsulated in ELP-PL drives the phase transition of ELP-PL, converting the PL to phase-separated ELP coacervate droplets.

### Photothermal phase transition of VO_x_@ELP-PL sequesters HSPs in cells

**Fig. 4.**
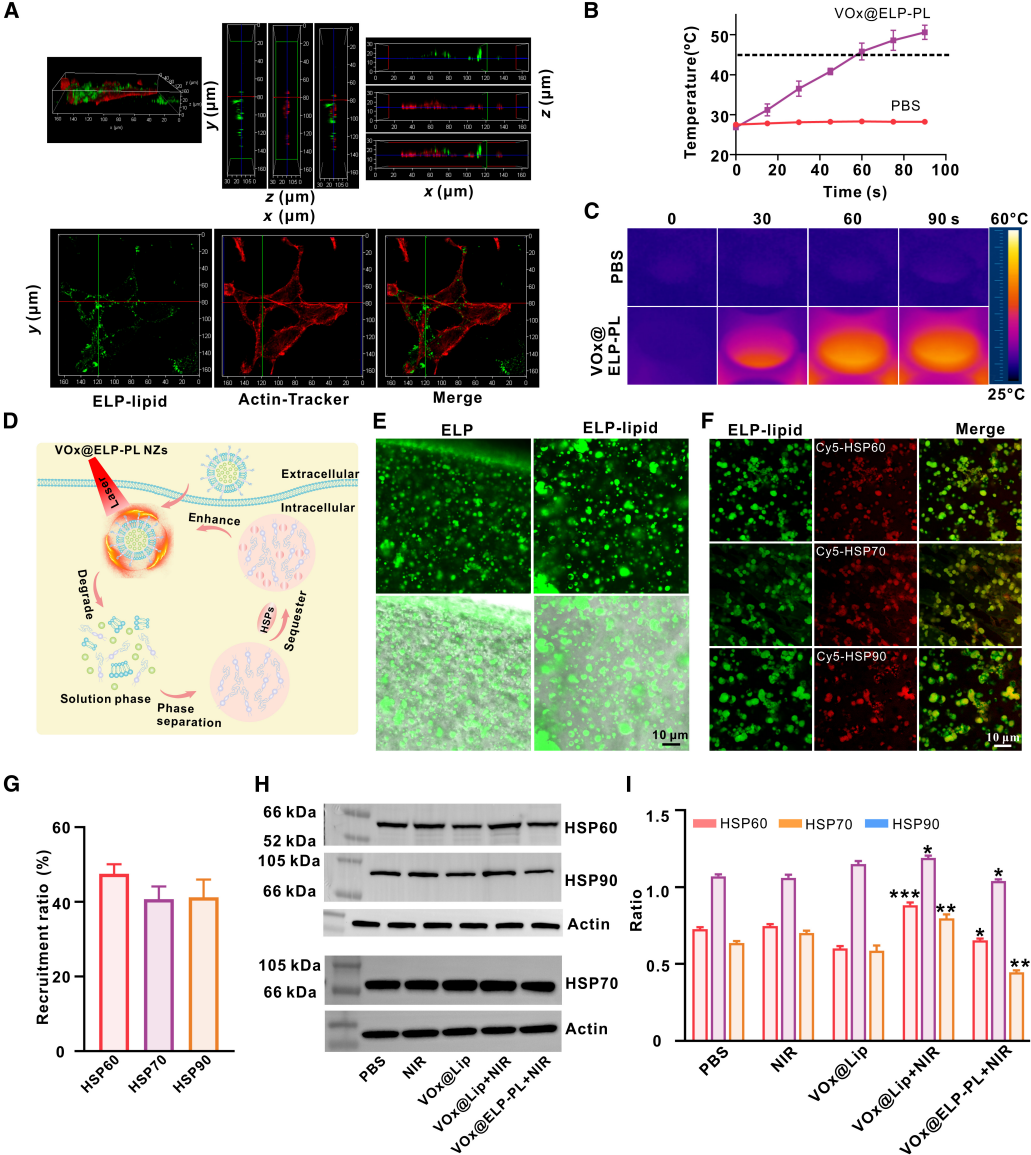
ELP coacervate droplets recruit HSPs in response to thermal effects in vitro. (A) 3D confocal images of CT26 cells incubated with VOx@ELP-PL (VOx: 1 mg/ml, ELP: 0.1 mg/ml) for 2 h (1 W/cm^2^, 1 min). 3D confocal z-stacks were acquired with a step size of 1 μm. Orthogonal views (*z* = 15 μm for the *xy* view, *y* = 80 μm for the *xz* view, and *x* = 120 μm for the *yz* view) were generated from the 3D volume using LAS X 3D software. (B and C) Infrared thermal imaging and the corresponding temperature changes of CT26 cells in a 96-well plate incubated with PBS and VO_x_@ELP-PL (VO_x_, 1 mg/ml; ELP, 0.1 mg/ml) for 2 h. The data are presented as mean ± standard deviation (*n* = 3 independent samples). (D) A schematic illustration showing ELP–lipid phase separation forms ELP coacervate droplets that recruit HSPs to enhance the photothermal therapy mechanism. The photothermal effect of VO_x_ changes the phase separation of ELP–lipid, recruiting thermal-induced HSPs and improving the photothermal therapy effect of VO_x_ NZs. (E) Fluorescent images of ELP (0.1 mg/ml) and ELP–lipid at 45 °C showing the formation of ELP coacervate droplets in vitro. The excitation and emission wavelengths were set at 488 and 525 nm, respectively. (F) Fluorescent images of ELP coacervate droplets showing the recruitment of Cy5-labeled HSPs (HSP: 0.1 mg/ml) and the corresponding recruitment efficiency. The excitation and emission wavelengths were set at 649 and 670 nm for the red channel, and 488 and 525 nm for the green channel. (G) Quantification of HSP recruitment in ELP coacervate droplets. Data are presented as mean ± standard deviation (*n* = 3 independent samples). (H) Western blotting (WB) analysis showing the cellular levels of HSPs treated by different VO_x_ NZ formulations in CT26 cells. Proteins in the soluble fraction (supernatant) of cell lysates were analyzed by Western blot. (I) Quantification of the WB image based on grayscale analysis. *P < 0.05; **P < 0.01; ***P < 0.001. The data are presented as mean ± standard deviation (*n* = 3 independent samples).

Subsequently, we demonstrated the photothermal effect of VO_x_@ELP-PL in cancer cells. Three-dimensional (3D) confocal images revealed, from both top and side views, that the ELP–lipid droplets were predominantly localized within the cellular cytoskeletal framework. This spatial distribution confirms the successful internalization of the PLs into the cells (Fig. 4A). Based on thermal imaging analysis, we showed that laser irradiation rapidly raised the temperature of CT26 cells treated with VO_x_@ELP-PL to about 45 °C within 1 min (Fig. 4B and C), above the critical temperature of ELP-PL to undergo phase transition. Under the confocal fluorescent microscope, VO_x_@ELP-PL initially showed dispersed fluorescent signals in CT26 cells. Photoillumination with an 808-nm laser induced the appearance of fluorescent puncta, consistent with the uptake of fluorescent coacervate droplets into cells (Fig. 4A). These data are consistent with the notion that photo-irradiation causes a thermal-responsive phase transition of VO_x_@ELP-PL in CT26 cells, from liposomes to phase-separated ELP coacervate droplets.

Next, we examined whether the ELP coacervate droplets could enrich and sequester HSPs that the cell overexpressed in response to the temperature rise (a scheme is shown in Fig. 4D). We further verified that the ELP–lipid conjugate could form coacervate droplets, similar to those of ELP, at elevated temperatures (Fig. 4E). ELP–lipid coacervate droplets enriched Cy5-labeled HSP60, HSP70, and HSP90 at 45 °C, indicated by the overlap of the red (Cy5) fluorescence and green fluorescence, with recruitment efficiencies measured to be around 40% (Fig. 4F and G). Isothermal titration calorimetry was then used to characterize the interactions between HSPs and ELP in 2 distinct states: as dispersed molecules and as coacervate droplets, respectively (Fig. S7). The results showed a state-dependent binding behavior. For water-soluble ELP, which was in a dissolved state, no marked heat change was detected upon injection of HSPs, suggesting the absence of specific high-affinity interactions between individual ELP and HSP molecules. In contrast, titration of HSPs into ELP coacervate droplets produced a pronounced heat signal, indicating substantial binding. These data demonstrated that phase-separated ELP coacervate droplets interact with HSPs, providing a physicochemical basis for their ability to encapsulate HSPs. To examine the HSP sequestering effect within cells, we conducted Western blotting (WB) analysis of HSP levels in CT26 cells. The results showed that, without laser irradiation, the expression of HSPs in the VO_x_@Lip-treated group was not significantly different from that in the phosphate buffer saline (PBS) group. After laser irradiation, the expression of all 3 HSPs, HSP60, HSP70, and HSP90, increased significantly, showing that the photothermal effect of VO_x_ led to thermal response in CT26 cells and caused elevation of HSP expression. Impressively, in the VO_x_@ELP-PL-treated group, the increase of HSP levels was significantly suppressed, suggesting that intracellular ELP-PL formed coacervate droplets that sequestered and reduced the level of soluble HSPs produced by the heat response in cells (Fig. 4H and I). Notably, during sample preparation for WB analysis, the coacervate droplets will be precipitated together with other cell debris during the centrifugation step, and only the soluble proteins in the supernatant will be analyzed by SDS-PAGE. These results provided evidence that VO_x_ NZs, when encapsulated in ELP-PL and delivered into cells, convert photons to thermal energy, thereby elevating the temperature of cells and inducing hyperthermia, as well as the overexpression of HSPs within cells. Subsequently, the temperature-induced phase transition of ELP-PL to coacervate droplets sequesters HSPs in situ and offsets the thermoresistant protective mechanism.

### Cell responses of VO_x_@ELP-PL treatment

**Fig. 5.**
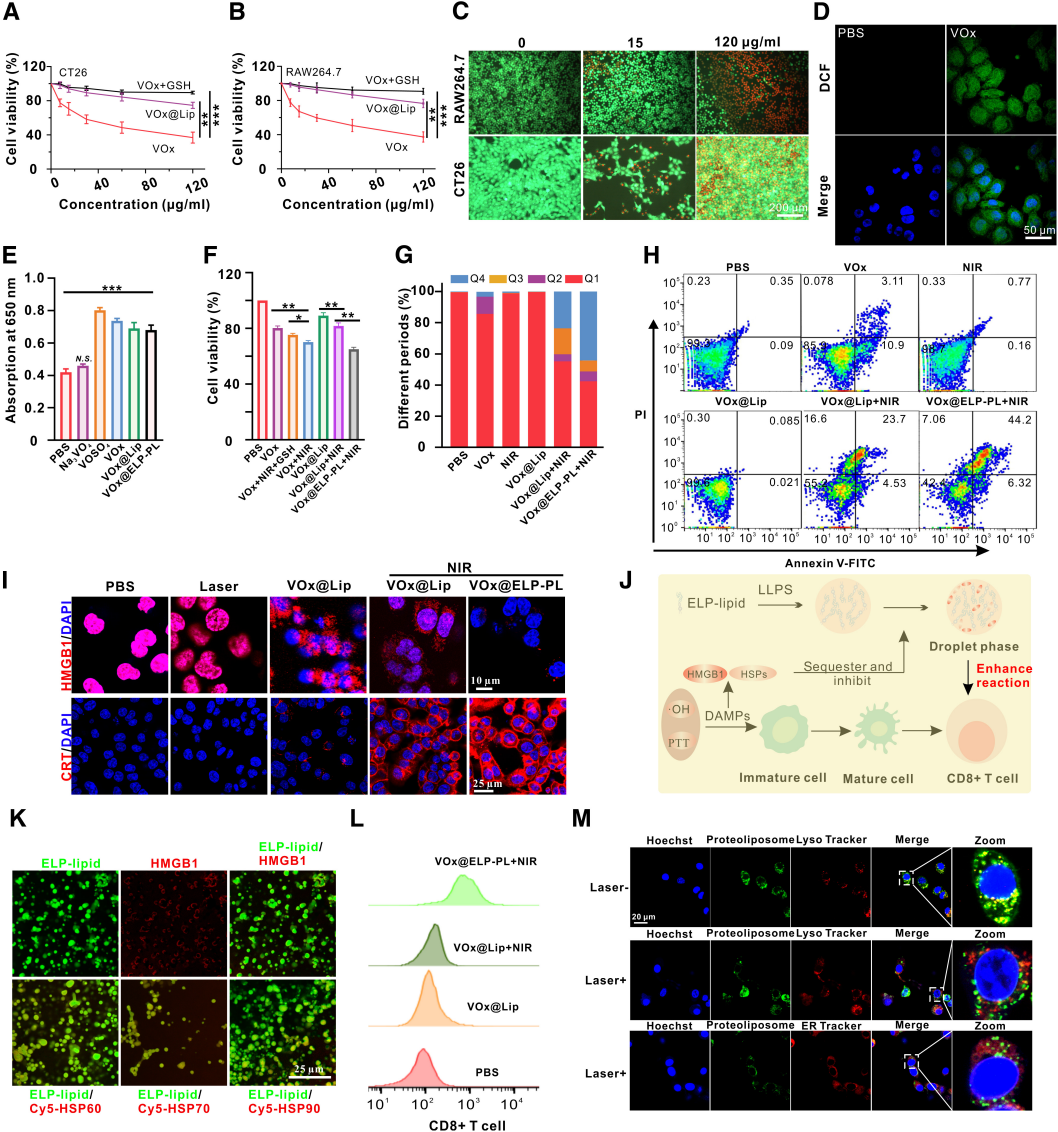
Cell responses induced by VO_x_@EPL-PL. (A and B) Cell Counting Kit-8 (CCK-8) toxicity assay after coincubation of CT26 and RAW264.7 cells with different concentrations of VO_x_ NZs and liposome-based NZs. Data are presented as mean ± standard deviation (*n* = 6 independent samples). (C) Calcein-PI staining fluorescence imaging after coincubation of CT26 and RAW264.7 cells with different concentrations of VO_x_ NZs. (D) 2,7-Dichlorodihydrofluorescein diacetate (DCFH-DA) detection of reactive oxygen species (ROS) in CT26 cells incubated with different probes. (E) The absorption value at 650 nm after adding H_2_O_2_ and tetramethylbenzidine (TMB) to different probes for 5 min. (F) Cell viability of CT26 cells after different treatments (VO_x_ concentration was 15 μg/ml, incubation time was 6 h). The data are presented as mean ± standard deviation (*n* = 6 independent samples). (G and H) Flow cytometric detection of apoptosis induction after coincubation of different probes (PBS, VO_x_, laser, VO_x_@Lip, VO_x_@Lip + laser, and VO_x_@ELP-PL + laser) with CT26 cells for 6 h and proportion statistics of different cell death periods. (I) Fluorescence analysis of the expression of HMGB1 and CRT in CT26 cells after different treatments. (J) Schematic diagram of the enhancement of tumor immune responses. Briefly, when VO_x_@ELP-PL degrades and releases ELP–lipid, ELP–lipid undergoes LLPS and partially recruits HMGB1 and HSPs both inside and outside the cells, thereby activating T cells. (K) Fluorescence images of ELP–lipid recruitment of different Cy5-labeled HSPs (0.1 mg/ml) and HMGB1 (0.1 mg/ml). The excitation and emission wavelengths were set at 649 and 670 nm for the red channel, and 488 and 525 nm for the green channel. (L) Flow cytometric detection of T cells after incubation with CT26 cells treated with different probes for 6 h. (M) Colocalization analysis of endoplasmic reticulum, lysosome, and mitochondria after VO_x_@ELP-PL coincubated with CT26 cells and irradiated with an 808-nm laser. A total of more than 6 cells from 3 biologically independent experiments were analyzed. The colocalization analyses were performed using ImageJ. For each image, regions of interest were manually outlined based on the cellular morphology. Background was subtracted by setting a rolling ball radius of 50 pixels. To determine the Pearson,s correlation coefficient, thresholds were automatically applied using the Costes, algorithm to exclude pixels with intensities below the background level. The excitation and emission wavelengths were set at 552 and 570 nm for the red channel, and 488 and 525 nm for the green channel.

Next, we evaluated the overall cell response during the photothermal treatment. VO_x_ NZs can be gradually etched into particles and vanadium ions of different valence states through redox reactions. High concentrations of vanadium compounds will cause cytotoxicity. Here, we found that as the concentration of VO_x_ NZs increased, the viability of CT26 and RAW264.7 cells gradually decreased (Fig. 5A and B). At 120 μg/ml, the survival rate of CT26 and RAW264.7 cells decreased to about 30%. Calcein and propidium iodide (PI) staining also showed that when the concentration of VO_x_ NZs reached 120 μg/ml, apoptotic cells (red) accounted for the majority of the cell population (Fig. 5C). However, encapsulating VO_x_ NZ in liposomes significantly protected cells against the toxicity of VO_x_ NZs, and cell viability in the VO_x_ + GSH group was also significantly higher than that in the VO_x_ alone group. This result further suggests that VO_x_ induces cell damage by generating reactive oxygen species (ROS) (Fig. 5A and B). At a VO_x_ NZ concentration of 120 μg/ml, the survival rates of CT26 and RAW cells increased to 74.6% and 76.6%, respectively. Because ion toxicity is most likely caused by highly toxic radicals [15], we probed the presence of ROS after the incubation of VO_x_ NZs and CT26 cells using 2,7-dichlorodihydrofluorescein diacetate (DCFH-DA). The results showed that compared with the PBS group, VO_x_ NZ-treated cells showed a markedly higher ROS level (Fig. 5D). VO_x_ exists in various valence states. According to our previous research [15], V^5+^ can generate V^4+^ under the reduction of GSH, and V^4+^ has strong peroxidase activity and can generate highly toxic •OH. Therefore, we further explored the peroxidase activity of the nanozyme. After the incubation of PBS, Na_3_VO_4_, VOSO_4_, VO_x_, VO_x_@Lip, and VO_x_@Lip-ELP with 1 mM of H_2_O_2_, and the addition of TMB (tetramethylbenzidine), the absorption value at 650 nm was measured (Fig. 5E). Compared with the PBS group, treatment with VOSO_4_, VO_x_, VO_x_@Lip, and VO_x_@Lip-ELP groups significantly increased the fluorescent signal, except Na_3_VO_4_. These results suggest that V^4+^ species may be primarily responsible for the peroxidase activity of VO_x_@ELP-PL, which may be a key source of the cytotoxicity to supplement the effect of hyperthermia. Cytotoxicity experiments also showed that NIR irradiation of VO_x_ NZ-treated CT26 cells caused an additional reduction in the cell survival rate, exemplifying the combination of metal and thermal cytotoxicity (Fig. 5F). For VO_x_@Lip, which showed slightly lower cytotoxicity due to the protection of the liposome, NIR irradiation caused a similar decrease in the survival rate. This can be explained by the fact that the photothermal effect of VO_x_ NZs destroyed the liposome structure, leading to the release of vanadium ions. The VO_x_ + NIR group exhibited a stronger cell-killing effect than the VO_x_ + NIR + GSH group, showing a combined therapeutic outcome attributable to both the photothermal effect of VO_x_ and its capacity to generate ROS. Comparing cells treated with VO_x_@EPL-PL and VO_x_@Lip, NIR irradiation caused a significantly higher cytotoxicity in the former group, possibly due to the phase transition property of the ELP-PL. To investigate the effect of HSPs on the observed cytotoxicity, we performed rescue experiments by delivering exogenous HSP60, HSP70, and HSP90 via liposomal carriers. The results showed that supplementation of these HSPs significantly increased cell viability compared to treatment with VO_x_@ELP-PL alone. This further confirms that HSPs can effectively mitigate the cytotoxic effects induced by PTT (Fig. S8). Flow cytometry analysis indicated that in VO_x_@Lip-treated cells, cell apoptosis was mainly in the late stage. However, in VO_x_@ELP-PL-treated cells, cell necrosis markedly increased (Fig. 5G and H). The combination of photothermal effect and hydroxyl radicals exerts a direct cell-killing effect and induces ICD. During this process, DAMPs are known to be released in large quantities, including HSPs, high mobility group box 1 (HMGB1), and calreticulin (CRT). We observed that hyperthermia and hydroxyl radicals induced the migration of HMGB1 from the nucleus to the cytoplasm, followed by extracellular secretion. Additionally, we observed a high expression of CRT on the CT26 cell membrane (Fig. 5I). ELP coacervate droplets could partially recruit HSPs and HMGB1 proteins (Fig. 5J). The fluorescence overlap of ELP coacervate droplets (green) with Cy5-labeled HMGB1, HSP60, HSP70, and HSP90 well demonstrated that ELP coacervate droplets could recruit HMGBs and HSPs (Fig. 5K and Fig. S9) and also inhibited the expression of HSPs (Fig. 4H) in vitro. After incubating CT26 cells treated differently with T cells and DC cells for 48 h, the flow cytometry results showed that the VO_x_@Lip-ELP + NIR group could achieve marked activation of CD8^+^ T cells, significantly different from the VO_x_@Lip + NIR group (Fig. 5L and Fig. S10). To evaluate the intracellular fate of PLs under NIR irradiation, we performed confocal colocalization studies before and after laser treatment. Without irradiation, PLs primarily colocalized with lysosomes, as indicated by a high Pearson’s correlation coefficient of 0.71. Following NIR treatment, photothermal heating disrupted the PL structure and released ELP–lipid components, which subsequently underwent LLPS to form intracellular coacervate droplets. These coacervate droplets exhibited markedly reduced colocalization with lysosomes, with a Pearson’s coefficient of 0.41, and showed no marked association with the endoplasmic reticulum, which was also confirmed by a 3D colocalization cell experiment (Fig. 5M and Fig. S11). These data show that the light-induced phase transition of VO_x_@EPL-PL elicits a range of responses within cancer cells and markedly enhances the efficacy of cancer treatment.

### The anticancer activity of VO_x_@ELP-PL in vivo

Lastly, we evaluated the anticancer activity of VO_x_@ELP-PL in an animal model of cancer. The safety of VO_x_@ELP-PL following intravenous injection was first evaluated using blood tests and biochemical analyses (Fig. S12). Twenty-five days after the injection of VO_x_@ELP-PL, white blood cell, red blood cell, platelet, and hemoglobin levels of mice were all at normal levels with no significant difference from control and animals on day 1 and day 0. Alanine aminotransferase and aspartate aminotransferase were also within the normal range. This showed that VO_x_@ELP-PL had no obvious toxicity to liver function. Hematoxylin and eosin (H&E) staining also showed that the morphology of each organ was intact, with no marked morphological abnormalities or inflammatory infiltration (Fig. S13). All the above indicate that VO_x_@ELP-PL is generally safe for the animal.

**Fig. 6.**
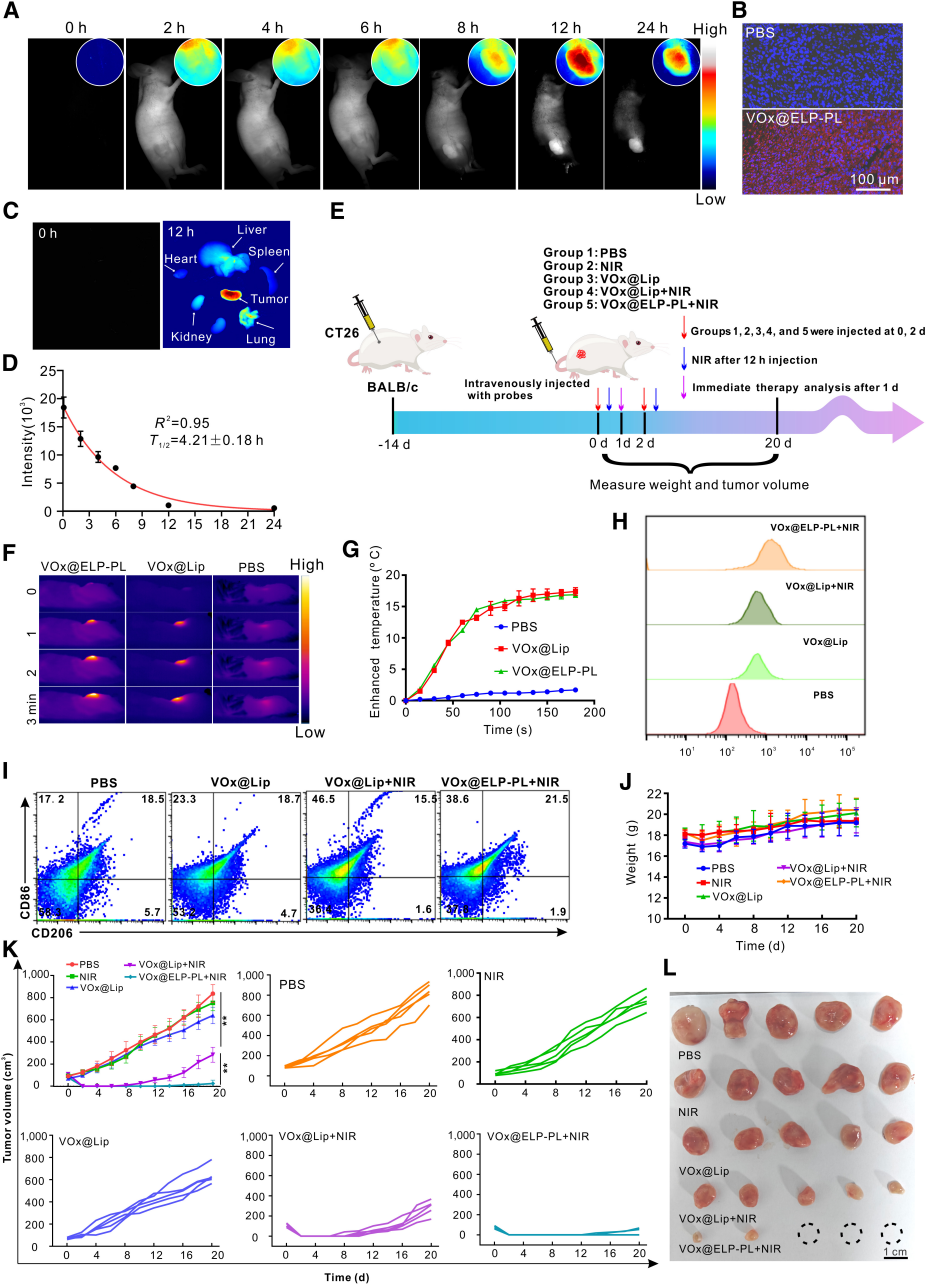
In vivo metabolism and antitumor efficacy of VO_x_@ELP-PL. (A) In vivo fluorescence imaging of VO_x_@ELP-PL labeled with mCherry in CT26 tumor-bearing mice. (B) Tissue sections of mice injected with PBS and mCherry-labeled VO_x_@ELP-PL 24 h later. (C) Fluorescence imaging of organs and tumors at 0 and 12 h after injection of mCherry-labeled VO_x_@ELP-PL. (D) Quantification of metabolic analysis of the probe in mice. Briefly, 200 μl of the VO_x_@ELP-PL labeled with mCherry (0.2 mg/ml) was injected into the tail vein, and the fluorescence intensity (670 nm/700 nm) of the localized tumor area was analyzed with ImageJ. (E) Schematic illustration showing the timeline of treatment for CT26 tumor-bearing mice with different probes. Briefly, 1 × 10^6^ CT26 cells were subcutaneously inoculated onto the back of BALB/c mice. When tumors grew to about 40 mm^3^, 200 μl of the materials (2 mg/ml of VO_x_) was injected through the tail vein. The above operation was performed daily and repeated 3 times. The tumor sizes were measured using vernier calipers. (F and G) Infrared thermal imaging and temperature change statistics of different probes injected into CT26 tumor-bearing mice irradiated with NIR laser. (H and I) Flow cytometry detection of macrophages and CD8^+^ T cells in tumor tissues after different treatments. (J to L) Body weight changes, tumor volume, and bright-field image of mice after different treatments. The data are presented as mean ± standard deviation (*n* = 5 independent samples).

A subcutaneous tumor model was established by inoculating CT26 colon cancer cells into mice. VO_x_@ELP-PL or other formulations were injected through the tail vein. Using mCherry-labeled ELP to track the injected VO_x_@ELP-PL in mice, we found that ELP-PL began to enrich at the tumor site 6 h after injection, reaching its maximum at 12 h, and then gradually decayed (Fig. 6A). Fluorescent images of the tumor slices dissected 24 h postinjection showed that VO_x_@ELP-PL was widely distributed in the tumor tissue and inside the colon cancer cells (Fig. 6B). Based on fluorescent images of the dissected organs, we also observed a markedly higher fluorescent signal in tumor tissue than in any other organs (Fig. 6C). Fluorescent imaging of mouse blood at different time points indicated that the VO_x_@ELP-PL had a long blood half-life (4.21 h) (Fig. 6D).

Subsequently, we intravenously injected different formulations into CT26 tumor-bearing mice over 2 consecutive days and observed tumor changes to evaluate in vivo antitumor efficacy (Fig. 6E). Thermal imaging analysis showed that laser irradiation increased the temperature of the mouse tumor by about 15 °C in the VO_x_@Lip and VO_x_@ELP-PL groups (Fig. 6F and G). It should be noted that the efficacy of the photothermal phase-transition mechanism may be influenced by intratumoral thermal heterogeneity in vivo, which could lead to variable local coacervate droplet formation and HSP recruitment across the tumor mass. Furthermore, we dissected the tumor tissues, dissociated the cells, and performed flow cytometry analysis. The results showed that a small number of CD8^+^ T cells were induced in the VO_x_@Lip group compared with the PBS group, possibly because the degradation of VO_x_ NZs promoted the production of •OH and induced an immune response. NIR irradiation led to a significant increase in CD8^+^ T cells in the tumor, as PTT further enhanced the immune effect (Fig. 6H and Fig. S14). Flow cytometry analysis of the VO_x_@ELP-PL treatment group showed a decrease in the number of proinflammatory macrophages (Fig. 6I and Fig. S15) and an increase in the number of CD8^+^ T cells (Fig. 6H). There was no significant difference in the weight of each group 20 d after the treatment (Fig. 6J), indicating a high tolerability and low toxicity of the preparations. In the 3 control groups (PBS, NIR, and VO_x_@Lip groups), tumor volumes gradually increased over time (Fig. 6K), suggesting that laser irradiation alone did not alter tumor volume and that encapsulating VO_x_ NZs in liposomes could effectively avoid the leakage of ion toxicity. As intravenous injection of VO_x_ NZs caused mouse death due to the acute toxicity, we changed to intratumoral injection of VO_x_ NZs. Intratumoral injection of VO_x_ NZs significantly reduced tumor size compared with the PBS group (Fig. S16A and B). H&E and terminal deoxynucleotidyl transferase-mediated deoxyuridine triphosphate nick end labeling (TUNEL) staining showed marked apoptosis and necrosis in the VO_x_ NZ group (Fig. S16C) and a high level of ROS, as confirmed by the DCFH-DA staining (Fig. S16D), suggesting that VO_x_ NZs in the tumor tissue could produce ROS and exhibit peroxidase activity. In contrast, in the VO_x_@Lip + NIR treatment group, the tumor volume decreased significantly compared with the VO_x_@Lip group, indicating that the photothermal effect of VO_x_ led to local tumor cell killing. Secondly, PTT disrupted the temperature-sensitive liposome, releasing vanadium ions that further synergistically killed tumor cells. The VO_x_@ELP-PL + NIR group showed the most effective tumor shrinkage among all the treatment groups (Fig. 6L), suggesting that the photothermal effect of VO_x_ NZs induces the phase transition of ELP-PL to ELP coacervate droplets to recruit HSPs, which promotes the PTT effect. At the end of treatment, 3 mice in the VO_x_@ELP-PL group did not experience tumor recurrence, suggesting a complete eradication of tumor cells.

### Phase transition of VO_x_@ELP-PL in vivo

**Fig. 7.**
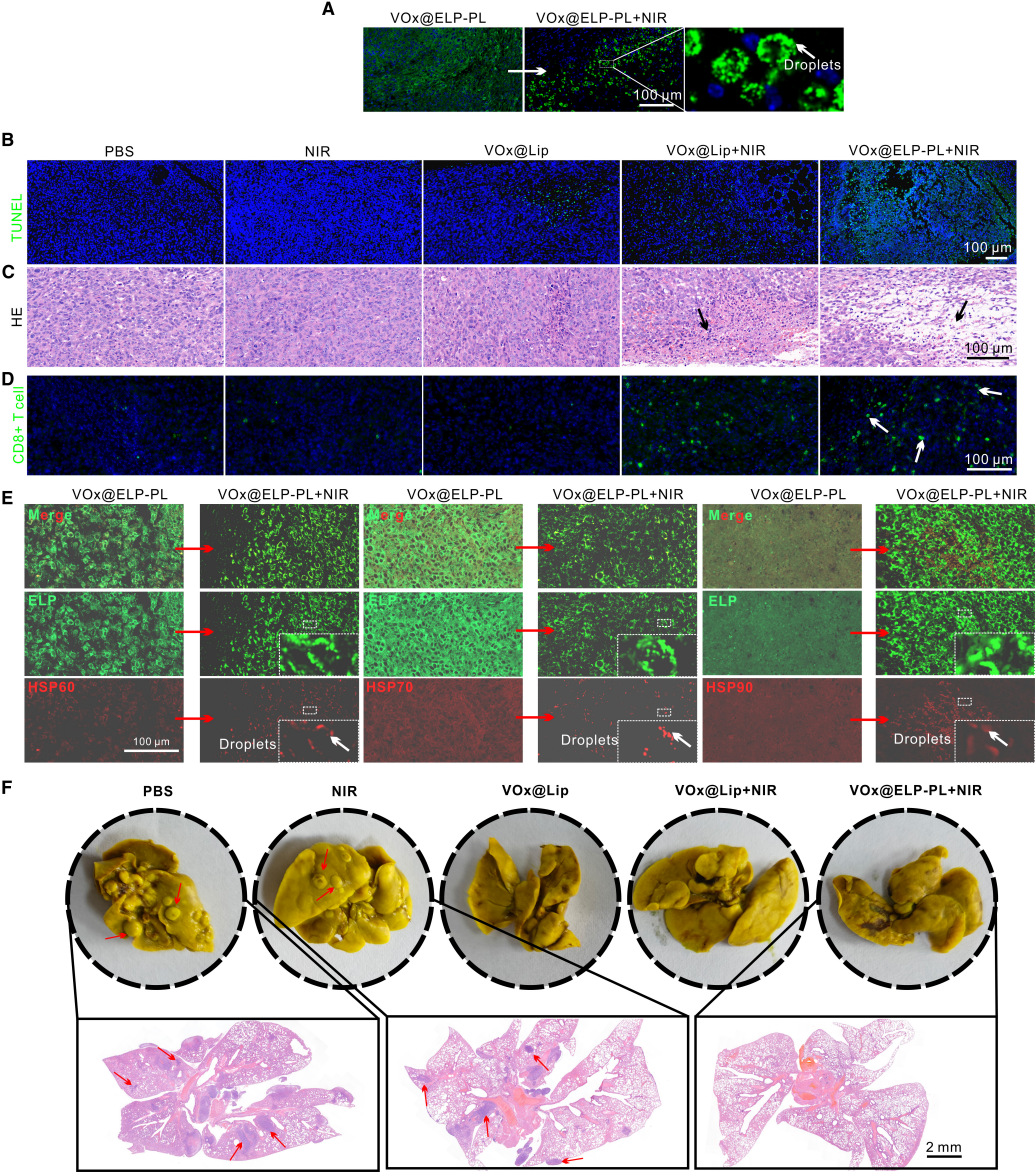
Morphological analysis of tumor tissue and organs. (A) Tumor sections of CT26 tumor-bearing mice injected with EGFP-labeled VO_x_@ELP-PL before and after laser irradiation. (B to D) Hematoxylin and eosin (H&E), terminal deoxynucleotidyl transferase–mediated deoxyuridine triphosphate nick end labeling (TUNEL), and CD8^+^ T cells staining of tumor sites in subcutaneous CT26 tumor-bearing mice treated with different probes and immunofluorescence staining of HSP 60, 70, and 90 proteins. Briefly, 1 day after the treatment, the mouse tumors were surgically removed, fixed with paraformaldehyde, embedded in paraffin, stained, and photographed. (E and F) Bouin’s fixation and H&E sections of lung tissues in different probe treatment groups after 20 d.

Subsequently, we studied the effect of the 808-nm laser on the phase transition of ELPs in vivo. The results showed that without laser treatment, VO_x_@ELP-PL diffused in tumor cells. After laser treatment, localized aggregated droplets appeared, which may be due to the phase separation of ELPs promoted by the photothermal effect of VO_x_ (Fig. 7A). The results of H&E staining showed that the nucleus and cytoplasm of cells in the PBS group were normal, while a certain amount of cell atrophy and cell separation occurred in the VO_x_@Lip + NIR group (Fig. 7B). In the VO_x_@ELP-PL + NIR group, this trend was markedly higher, along with the TUNEL staining data, further confirming the effective tumor-killing effect of VO_x_@ELP-PL (Fig. 7C). Moreover, the immunofluorescence imaging results indicated that the CD8^+^ T cell induction level was also the highest in the VO_x_@ELP-PL + NIR group (Fig. 7D). These data confirm that the VO_x_@ELP-PL + NIR treatment induced marked tumor cell killing in vivo. We also found that before irradiation, ELP, HSP60, HSP70, and HSP90 were diffusely distributed in the cells; while after irradiation, their distribution became locally granular. More importantly, there was substantial overlap between the fluorescence of ELP and HSPs, suggesting that ELP could recruit HSPs in vivo (Fig. 7E). Altogether, these data confirmed that VO_x_@ELP-PL could enhance the PTT effect of VO_x_ and achieve synergistic tumor cell killing. Moreover, upon lung dissection, a large number of lung nodules were found in the PBS, NIR, and VO_x_@Lip groups (Fig. 7F). The presence of these cancer stem cells (CRC) in the nodule area indicated that CRC cells had metastasized to the lungs. In contrast, metastasis to the lung was not observed in the VO_x_@ELP-PL treatment group, suggesting that VO_x_@ELP-PL inhibits tumor cell metastasis.

## Discussion

In this study, we synthesized a composite PL system, VO_x_@ELP-PL, consisting of liposomes formed by a phospholipid conjugate of an ELP (ELP–lipid) with VO_x_ NZs incorporated in the lumen. VO_x_ NZs possessed peroxidase activity and could catalyze hydrogen peroxide in tumors to produce highly toxic hydroxyl radicals. In addition, under the photothermal effect of VO_x_ NZs, the PLs degraded and released ELP–lipid, which undergoes a phase transition to form coacervate droplets above a critical temperature of 45 °C. The phase-separated coacervate droplets recruit HSPs produced by heat resistance, thereby enhancing the PTT of VO_x_ NZs and synergizing with the catalytic activity of nanozymes to inhibit tumor cell growth. More importantly, the combination of PTT and •OH caused cell necrosis, leading to the release of large amounts of DAMPs, including HSPs, HMGB, and others. We speculate that ELP coacervate droplets may modulate the local immune microenvironment, presumably by attenuating excessive infiltration and activation of CD8^+^ T cells and reducing the levels of key proinflammatory cytokines. This immunomodulatory effect contributes to the observed therapeutic outcomes and suggests a potential mechanism for mitigating inflammation-driven pathology. Taken together, we demonstrate that the composite PL system, VO_x_@ELP-PL, utilizes the phase transition of PL to coacervate droplets to sequester proteins in cells. This strategy employs a unique mechanism to amplify the efficacy of the photothermal agent, VO_x_ NZs, and augment PPT’s efficacy. Additionally, it is important to highlight that the therapeutic effect results from the interplay of photothermal triggering, LLPS-driven HSP sequestration, and concurrent ROS effects, rather than a single pathway. To our knowledge, this PL system represents one of the first coacervate droplet-mediated therapies. Admittedly, despite the absence of acute short-term toxicity, the long-term fate of VO_x_@ELP-PL in vivo remains to be investigated. Future studies focusing on the chronic pharmacokinetics, potential organ accumulation over extended periods, and detailed clearance pathways of vanadium species will be essential to fully translate this promising platform toward clinical application.

## Materials and Methods

All reagents and solvents were purchased without further purification. Vanadium pentoxide, sodium orthovanadate, vanadyl sulfate, and 3,3′,5,5′-tetramethylbenzidine were purchased from Shanghai Macklin Biochemical Co., Ltd. SDS was purchased from Bio-RAD. Amino-PEG-phospholipid (DSPE-mPEG_2000_-NH_2_), 1,2-distearoyl-sn-glycero-3-phosphocholine (DSPC), and 1,2-dipalmitoyl-sn-glycero-3-phosphocholine (DPPC) were purchased from Xi’an Ruixi Biotechnology Co., Ltd. The Annexin V-FITC (fluorescein isothiocyanate) apoptosis detection kit was purchased from Tongren Chemical Research Institute (Dojindo Laboratories); ER-Tracker Red, Lyso-Tracker Red, and DCFH-DA were purchased from Beyotime Biotechnology. Plasmid extraction was performed using Takara Plasmid Miniprep Kit or E.Z.N.A. Plasmid Mini Kit I, (V-spin) from Omega Bio-tek company. Cell Counting Kit-8 (CCK-8) was purchased from Med Chem Express. Five-week-old BALB/c mice (SPF grade) were purchased from Hunan Silaikejingda Experimental Animal Co., Ltd. Mice were housed in an animal facility under constant environmental conditions (room temperature, 22 ± 1 °C; relative humidity, 40% to 70%; and a 12-h light–dark cycle), and all mice had access to food and water ad libitum.

### Plasmid construction, expression, and purification of protein

One plasmid (SpyTag-Elp-CarH-Elp-SpyTag) was used in this study. *Escherichia coli* TOP10 was used for cloning and plasmid propagation and grown in selective Luria–Bertani medium or Luria–Bertani plates with 1.5 wt % agar. Ampicillin (100 μg/ml) was added for selection. The DNA sequences of the plasmid constructs containing PCR fragments were confirmed by sequencing. Recombinant plasmids were transformed into *E. coli* BL21 (DE3) for protein expression. Seed culture was prepared by picking a single colony into 3 ml of LB containing the corresponding antibiotics and shaking at 37 °C overnight. Then, 1% (v/v) overnight culture was added into sterilized terrific broth medium at 37 °C with 220 rpm until the optical density (OD) value reached 0.6 to 0.8. Isopropyl-β-D-1-thiogalactopyranoside (IPTG) was added to induce the protein expression. Afterward, cultures were incubated at 17 °C and 180 rpm overnight for protein expression. The cells were harvested by centrifugation (4,000 *g* for 10 min), and the pellets were washed twice with buffer and then frozen at −80 °C for the following purification. SpyTagELPEGFPELPSpyTag was bonded with a HiTrap His column for further purification. SDS-PAGE was used to assess protein expression and purification, and protein content was determined using a bicinchoninic acid kit with bovine serum albumin as the standard.

For protein purification, the cell pellets were suspended in lysis buffer (0.1 M PBS, pH 7.4, containing 1 M NaCl and 20 mM imidazole) and lysed by sonication for 30 min on ice. After centrifuging at 12,000 *g* for 30 min, the supernatant was filtered through a 0.22-μm filter and applied to a nickel column for 1 h. The purification process was conducted using fast protein liquid chromatography, and the protein was eluted with a step gradient of the elution buffer (0.1 M PBS buffer, pH 7.4, containing 1 M NaCl and 500 mM imidazole). Then, the eluted proteins were collected and concentrated using a 10-kDa membrane from Millipore, and then buffer-exchanged into storage buffer (0.1 M PBS buffer, pH 7.4, 150 mM NaCl) and frozen for storage at −80 °C.

### Preparation and characterization of ELP PL NZs (VO_x_@ELP-PL)

VO_x_ NZs were synthesized slightly modified according to the literature. V_2_O_5_ (13 mg/ml, 20 ml) and citrate (29 mg/ml, 10 ml) were mixed at room temperature. Then, NaBH_4_ (42 mg/ml, 10 ml) was injected. After reaction for 4 h, the product was centrifuged (11,000 rpm, 10 min) to collect the precipitate, and centrifuged with 2:1 acetone and ultrapure water twice. Finally, the precipitate was washed and dispersed in PBS to obtain VO_x_ NZs.

For ELP–lipid, ELP (2 mg/ml, 1 ml) was prepared in PBS (pH 8.0), and 10 molar equivalents of DSPE-PEG_2000_-NHS were added to react with ELP; the reaction was carried out at room temperature for 2 h. The unreacted raw materials were removed using a desalting column.

For ELP-modified liposome-based NZs, DPPC/DSPC/ELP–lipid was mixed at a mass ratio of 30:10:1, and then trichloromethane (15 ml) was added to a 25-ml round-bottom flask. The organic solvent was removed by vacuum rotary evaporation to prepare the lipid film, and hydrated by adding VO_x_ (2 mg/ml, 1 ml), then the hydrated liposome was extruded by Avanti micro extruder (200 nm polycarbonate porous membrane, 20 times) and centrifuged (11363 g, 10 min, 2 times) to prepare ELP-modified liposome-based nanozymes. The same method was used to obtain VO_x_@Lip.

The size and morphology of the samples were characterized by TEM (HT7700, Japan) and Nano-ZS90 zeta sizer (Malvern, UK). The absorption spectrum was measured with a UV-2550 UV–visible spectrophotometer (Shimadzu, Japan); the EDS spectrum was acquired using a Tecnai G20 U-Twin transmission electron microscope (FEI, Netherlands). Element valence analysis was obtained through AXIS-ULTRA DLD-600W x-ray photoelectron spectrometer (Shimadzu, Japan).

### ELP–lipid phase separation regulated by the photothermal effect of VO_x_ NZs

For the photothermal effect of VO_x_ NZs, 300 μl of VO_x_ NZs at different concentrations (0, 0.125, 0.25, 0.5, and 1 mg/ml) were irradiated with an 808-nm laser (1 W/cm^2^, 5 min), and the temperature was recorded by an infrared thermal imager. Similarly, 1 mg/ml of VO_x_ NZs was irradiated with the 808-nm laser under different powers (0.5, 1, 1.5, and 2 W/cm^2^), and the temperature change was also recorded. Each experiment was repeated 3 times. For the photo-stability of VO_x_, 300 μl of VO_x_ (1 mg/ml) was irradiated with NIR (808 nm, 2 W/cm^2^) for 5 min and stopped for 10 min. Then, an additional 4 NIR on/off cycles were repeated, and the temperature change was recorded. For the photothermal conversion efficiency of VO_x_, the absorbance of VO_x_ at 808 nm was measured, 300 μl of VO_x_ in a quartz cuvette was irradiated with NIR (2 W/cm^2^, 5 min), and the temperature change was recorded until the solution reached a steady-state temperature. The photothermal conversion efficiency was calculated with the equation, $\eta =\frac{\mathrm{hS}\left(T_{\mathrm{Max}}-T_{\text{Surr}}\right)-Q_{\mathrm{Dis}}}{I\left(1-10^{-A_{808}}\right)}$, with *h* representing the heat transfer coefficient; *S* denoting the surface area of the container; *T*_max_ and *T*_surr_ indicating the maximum and starting temperature, respectively; *Q*_Dis_ representing the energy emitted by the solvent; *I* denoting the NIR intensity; and *A*_808_ indicating the absorbance of VO_x_ at 808 nm. For the photothermal properties, VO_x_, VO_x_@Lip, and VO_x_@ELP-PL were irradiated with an 808-nm laser for 5 min, and the temperature changes were statistically analyzed.

For photothermal-induced ELP phase separation, VO_x_@ELP-PL (VO_x_: 2 mg/ml; ELP: 1 mg/ml) was irradiated with the 808-nm laser for 1 min, and the fluorescence images after irradiation were observed under a confocal microscope. For photothermal-induced ELP phase separation in CT26 cells, 50 μl of VO_x_@ELP-PL (VO_x_: 2 mg/ml; ELP: 1 mg/ml) was incubated with CT26 cells for 2 h. After washing twice with PBS, the cells were exposed to the 808-nm laser for 1.5 min. The temperature changes were recorded, and the CT26 cells were observed before and after laser irradiation under a confocal microscope.

### ELP–lipid phase separation recruits different HSPs

For the phase separation of phospholipid-ELP, 2.5 μl of ELP and ELP–lipid (1 mg/ml) was heated at 45 °C for 1 min, and then observed under a confocal microscope. Subsequently, we conducted the recruiting experiment of HSPs by ELP–lipid. First, Cy5-NHS was coupled to HSPs 60, 70, and 90, and the conjugates were purified using a desalting column. Then, 10 μl of ELP–lipid (1 mg/ml) and an equal amount of Cy5-labeled HSPs were mixed evenly, heated at 45 °C for 1 min, and centrifuged at 376 *g* for 5 min; then, the supernatant was discarded, and the precipitate was dissolved with PBS (2.5 μl) and observed under a confocal microscope. The unrecruited HSPs in the supernatant were detected by Bradford analysis, and the recruitment efficiency of HSPs by ELP–lipid was calculated.

About 5 × 10^5^ CT26 cells were inoculated in a 6-well culture plate and cultured in a 5% CO_2_ incubator at 37 °C for 12 h. The culture medium was then removed and treated with PBS, laser, VO_x_@Lip, VO_x_@Lip + laser, and VO_x_@ELP-PL + laser, respectively. Then, they were added to the protein extraction solution to collect and lyse the cells. The proteins were separated by 10% SDS-PAGE and then electrotransferred to a polyvinylidene fluoride membrane. To prevent the nonspecific binding of the antibody, the membrane was blocked in 5% skim milk powder (prepared in TBST) at 37 °C for 30 min, incubated with the primary antibody, washed with TBST 3 times at 4 °C overnight, and then incubated with the secondary antibody at room temperature for 30 min. Actin was used as a control. The film was scanned and archived, and the optical density value of the target band was analyzed by the Alpha software processing system.

### Cell apoptosis

For cytotoxicity, CT26 and RAW264.7 cells in the logarithmic growth phase were inoculated into 96-well plates and cultured overnight in an incubator (37 °C, 5% CO_2_). After removing the culture medium, serum-free culture medium (200 μl) containing different concentrations of VO_x_ (0, 3.8, 7.5, 15, 30, 60, and 120 μg/ml) and VO_x_@Lip (0, 3.8, 7.5, 15, 30, 60, and 120 μg/ml) was added. Six parallel wells were set up in each group. After 12 h, the culture medium was removed, and the cells were washed 3 times with PBS. Fresh serum-free culture medium (200 μl) was added, and the CCK-8 (20 μl) kit was added to each well. After another 3 h of incubation, the absorbance at 450 nm was measured using a microplate reader to calculate the cell survival rate. For live–dead staining of apoptosis, different concentrations of VO_x_ (0, 15, and 120 μg/ml) were incubated with CT26 and RAW264.7 cells in the logarithmic growth phase for 6 h, and the cells were washed 3 times with PBS. Calcein (5 μl, 10 min) and PI staining solution (10 μl, 10 min) were added. After washing 3 times, the cells were observed under a confocal microscope. Similarly, CT26 cells were cultured and treated with PBS, VO_x_ (10 μg/ml), laser, VO_x_@Lip, VO_x_@Lip + laser, and VO_x_@ELP-PL + laser, and after 6 h, Annexin V-FITC/PI apoptosis staining solution was added. After 15 min, the staining solution was removed, and the cells were washed with PBS 3 times before flow cytometry analysis of apoptosis.

For the phase separation of ELP–lipid at different temperatures, 50 μl of ELP–lipid (1 mg/ml) was incubated in a 384-well plate for the turbidity test (37, 40, 45, and 50 °C) and then observed under a confocal microscope. For the colocalization of ELP–lipid and organelles after photothermal induction, CT26 cells were cultured under the same conditions, and VO_x_@ELP-PL was added for 2 h. After irradiation with laser (808 nm, 0.5 W) for 1 min, probes for lysosome, endoplasmic reticulum, and mitochondria, and Hoechst 33342 were added, and after thoroughly rinsing with PBS, the cells were observed under a confocal microscope.

### In vivo antitumor activity

For the toxicity of VO_x_@ELP-PL in vivo, 15 5-week-old male BALB/c mice (SPF grade) were randomly divided into 3 groups, 2 of these groups were killed 1 day after tail vein injection of 200 μl of VO_x_@ELP-PL (2 mg/ml of VO_x_) and PBS, while the third group was killed in 25 d after injection of VO_x_@ELP-PL. Blood was collected for routine blood and biochemical analyses, and one mouse from each group was used for H&E staining.

Approximately 1 × 10^6^ CT26 cells were subcutaneously inoculated into 5-week-old male BALB/c mice (SPF grade). After 14 d, 200 μl of the VO_x_@ELP-PL (2 mg/ml of VO_x_) labeled with mCherry was intravenously injected for continuous fluorescence imaging at different time points (0, 2, 4, 6, 8, 12, and 24 h). Additionally, after 24 h, the mouse tumor-embedded sections were imaged using self-luminous mCherry scanning. Lastly, the mice were injected with VO_x_@ELP-PL (2 mg/ml of VO_x_) labeled with mCherry at different time points (0, 2, 4, 6, 8, 12, and 24 h), and blood was collected to measure fluorescence intensity and analyze the half-life of VOx@ELP-PL in blood.

Thirty BALB/c mice subcutaneously inoculated with CT26 cells were divided into 5 groups, and treated with (a) PBS, (b) NIR laser, (c) VO_x_@Lip, (d) VO_x_@Lip + laser, and (e) VO_x_@ELP-PL + laser (all the drugs were injected by the tail vein, and all the injections were repeated 2 times every 2 d). After the first injection, one mouse was taken from each group. In the laser treatment group, temperature changes were recorded using an infrared thermal imager, and H&E, TUNEL, HSP60, HSP70, and HSP90 immunofluorescence staining and autofluorescence were detected under the microscope at 12 h. For the remaining mice, the second injection was administered, and changes in weight and tumor volume were recorded every 2 d using digital vernier calipers and weight scales. After 20 d, the mice were sacrificed, and the lungs were removed, fixed in Bouin’s, and analyzed by H&E staining.

### Statistical analysis

All experiments were performed with at least 3 replicates. All values are presented as means ± SD. Bar and line charts were generated with GraphPad Prism (ver. 8.3.0). Comparisons between 2 groups were performed using a 2-tailed Student *t* test. Values with *P* < 0.05 are considered significant.

## Ethical Approval

All animal experiments were approved by the Animal Experiment Ethics Committee of Huazhong University of Science and Technology (IACUC number: 4207).

## Data Availability

The authors declare that the main data supporting the findings of this study are available within the article and its Supplementary Materials or from the authors upon request.
