# Supplementary material for: Photothermal-Responsive Phase Transition of Proteoliposomes for Heat Shock Protein Sequestering against Cancer Thermoresistance
Source: Research (Wash D C). 2026 Mar 31;9:1231. doi: 10.34133/research.1231 (PMC13036321; doi:10.34133/research.1231)
Supplement: Supplementary 1 — Figs. S1 to S16 [file research.1231.f1.pdf]

Supplementary Materials for

Photothermal-Responsive Phase Transition of Proteoliposomes for Heat Shock Protein Sequestering against Cancer Thermoresistance

The PDF file includes:

Figs. S1 to S16

Contents

Item	Page No.
**Figure S1.** The SDS-PAGE showing the purification of ELP.	**S3**
**Figure S2.** EDS energy spectrum of VOx@ELP-PL.	**S4**
**Figure S3.** Polymer dispersity index of VOx, VOx@Lip, VOx@ELP, and VOx@ELP-PL.	**S5**
**Figure S4.** Differential scanning calorimetry of liposome, ELP-PL, and VOx@ELP-PL.	**S6**
**Figure S5.** Turbidity at 600 nm of ELP-lipid at different temperatures.	**S7**
**Figure S6.** Phase-transition behavior of ELP and ELP-lipid during continuous heating	**S8**
**Figure S7.** Isothermal titration calorimetry analysis of ELP molecules and ELP coacervate droplets with different heat shock proteins.	**S9**
**Figure S8.** Cytotoxicity-rescue experiments in CT26 cells following different treatments	**S10**
**Figure S9.** Fluorescence imaging of different Cy5-labeled HSPs in ELP-lipid coacervate droplets.	**S11**
**Figure S10.** Quantitative flow cytometry analysis of $CD8^+$ T cells.	**S12**
**Figure S11.** 3D confocal microscope images to show that NIR laser irradiation significantly reduced colocalization of PL droplets with lysosomes.	**S13**
**Figure S12.** Counts of RBC, WBC, PLT, and HGB, and analysis of liver function indicators ALT and AST of animals following treatments.	**S15**
**Figure S13.** H&E staining of majors organs of animals following treatments.	**S16**
**Figure S14.** Quantitative flow cytometry analysis of $CD8^+$ T cells after different treatments *in vivo*.	**S17**
**Figure S15.** Quantitative flow cytometry analysis of macrophages after different treatments *in vivo*.	**S18**
**Figure S16.** Anti-tumor efficacy of PBS and VOx in mice bearing subcutaneous CT26 tumors.	**S19**

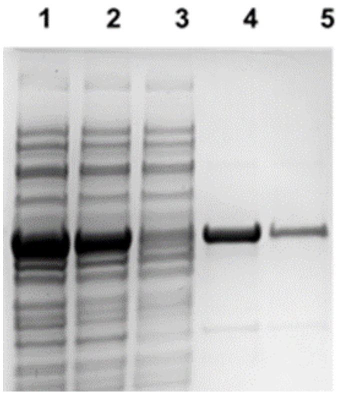

Figure S1. The SDS-PAGE showing the purification of ELP. Lane 1: cell lysate; lane 2: supernatant; lane 3: flow through; lane 4: elution of 5%; lane 5: elution of 100%.

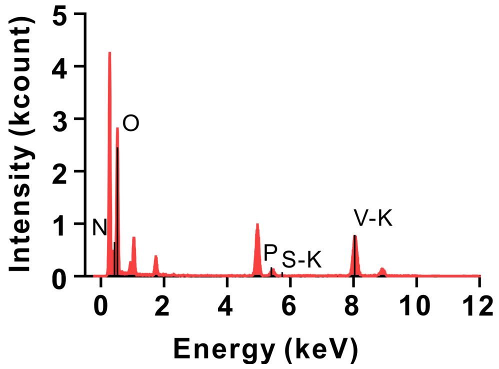

Figure S2. EDS energy spectrum of VO_x@ELP-PL.

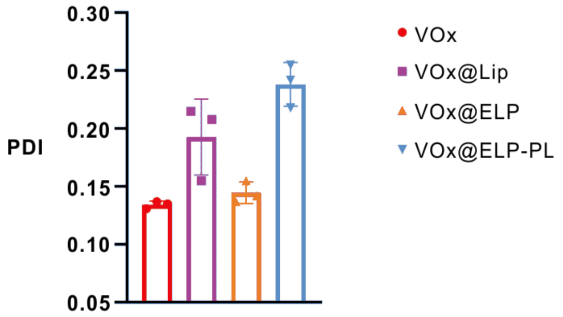

Figure S3. Polymer dispersity index (PDI) of VO_x, VO_x@Lip, VO_x@ELP, and VO_x@ELP-PL.

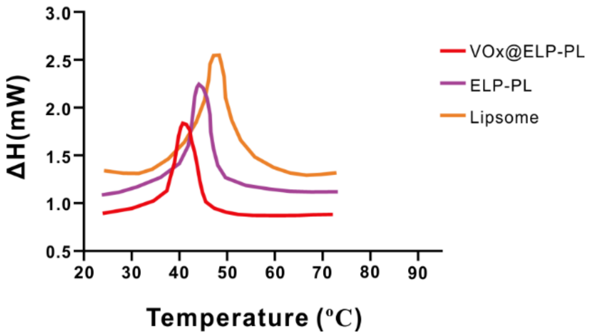

Figure S4. Differential scanning calorimetry (DSC) of liposome, ELP-PL, and VO_x@ELP-PL.

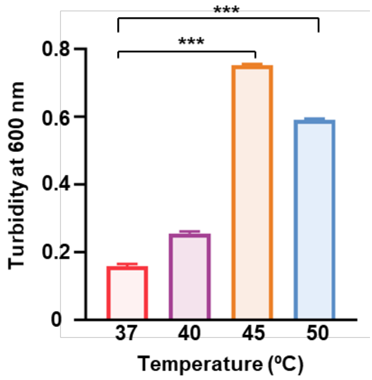

Figure S5. Turbidity at 600 nm of ELP-lipid at different temperatures (37, 40, 45, and 50 ºC).

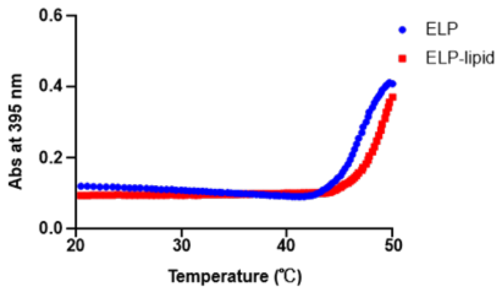

Figure S6. Phase-transition behavior of ELP and ELP-lipid during continuous heating. ELP or ELP-lipid, 0.5 mg/mL.

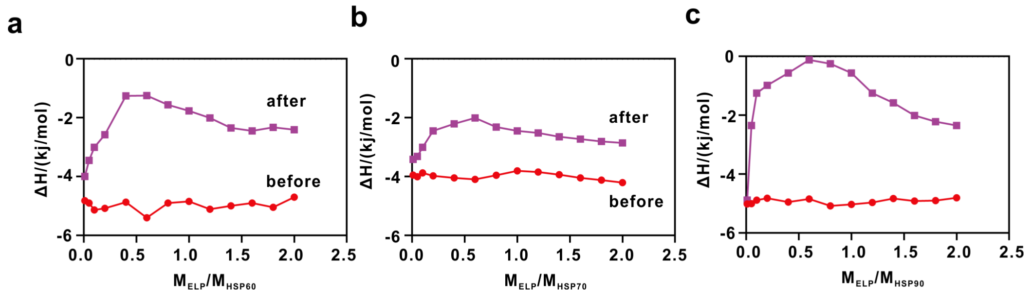

Figure S7. Isothermal titration calorimetry analysis of ELP molecules and ELP coacervate droplets with different heat shock proteins. **a**, HSP60; **b**, HSP70; **c**, HSP90.

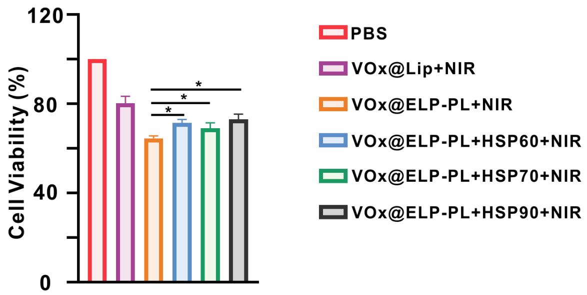

Figure S8. Cytotoxicity-rescue experiments in CT26 cells following different treatments. VO_x, 15 µg/mL; HSP, 100 µg/mL; incubation time, 6 h. The data are presented as mean ± standard deviation (n = 6 independent samples).

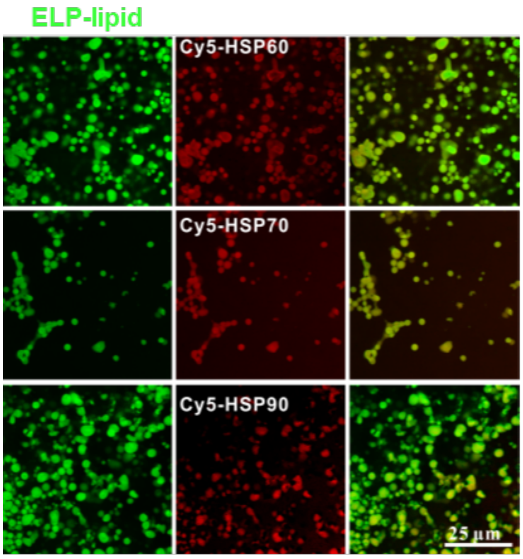

Figure S9. Fluorescence imaging of different Cy5-labeled HSPs in ELP-lipid coacervate droplets. HSP, 0.1 mg/mL. The excitation and emission wavelengths were set at 649 nm and 670 nm for the red channel, and 488 nm and 525 nm for the green channel.

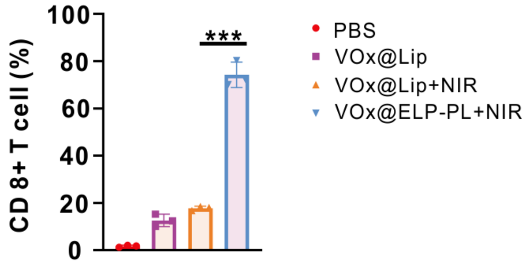

Figure S10. Quantitative flow cytometry analysis of $CD8^+$ T cells.

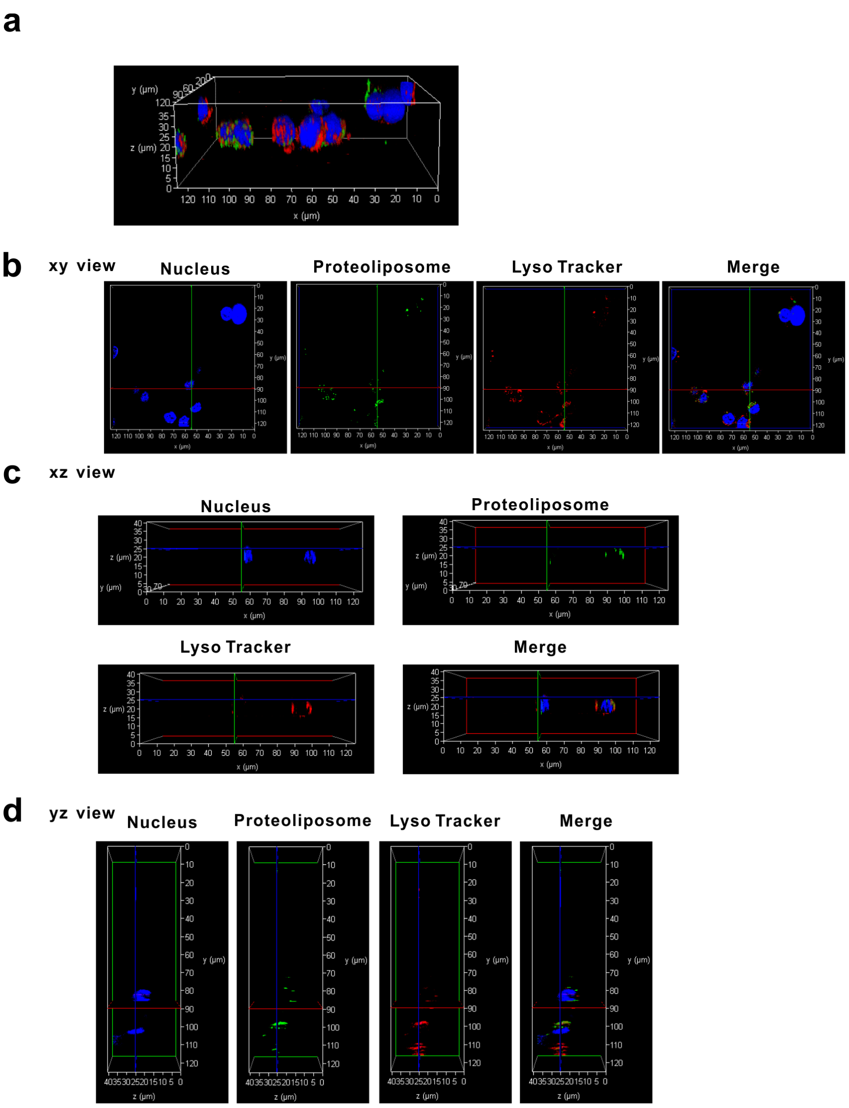

Figure S11. 3D confocal microscope images to show that NIR laser irradiation significantly reduced colocalization of PL droplets with lysosomes. The excitation and emission wavelengths were set at 552 nm and 570 nm for the red channel, and 488 nm and 525 nm for the green channel. 3D confocal z-stacks were acquired with a step size of 1 µm. Orthogonal views (z =15 µm for the xy view, y = 80 µm for the xz view, and x = 120 µm for the yz view) were generated from the 3D volume using LAS X 3D software. A total of more than 6 cells from three biologically independent experiments were analyzed. The colocalization analyses were performed using ImageJ. For each image, regions of interest were manually outlined based on the cellular morphology. The background was subtracted using a rolling ball radius

of 50 pixels. To determine the Pearson's correlation coefficient, thresholds were automatically applied using the Costes' algorithm to exclude pixels with intensities below the background level.

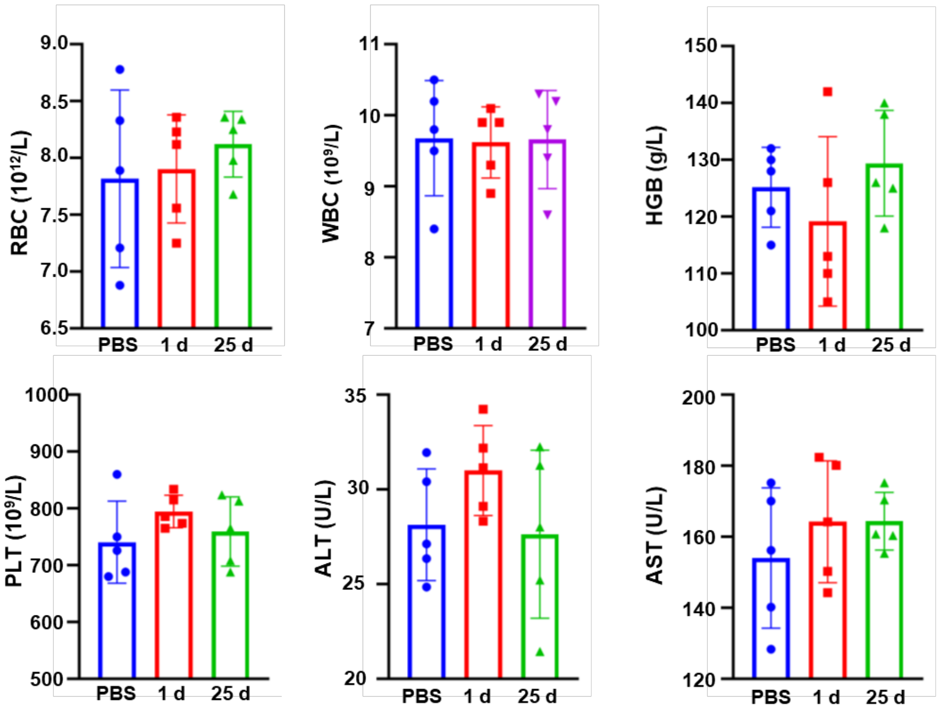

Figure S12. Counts of RBC, WBC, PLT, and HGB, and analysis of liver function indicators ALT and AST of animals following treatments. Analysis was taken 1 day or 25 days after the injection of VO_x@ELP-PL.

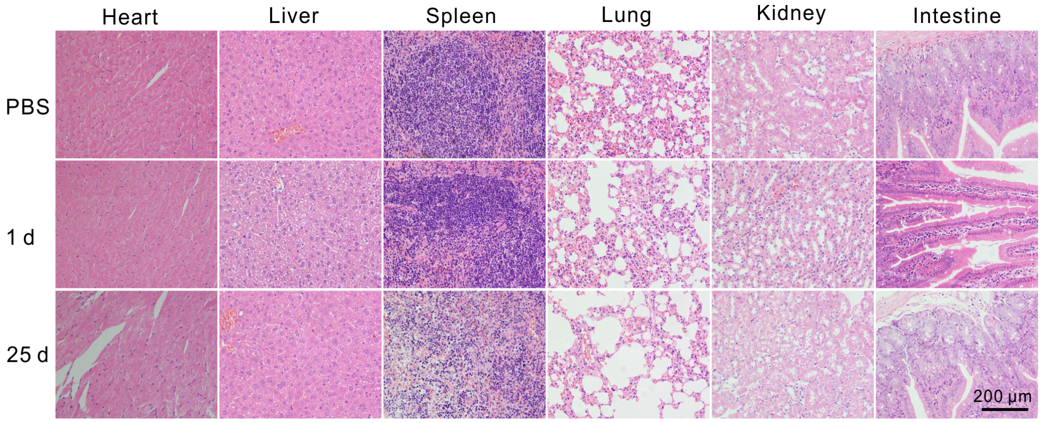

Figure S13. H&E staining of major organs of animals following treatments. Analysis was taken 1 day or 25 days after the injection of VO_x@ELP-PL.

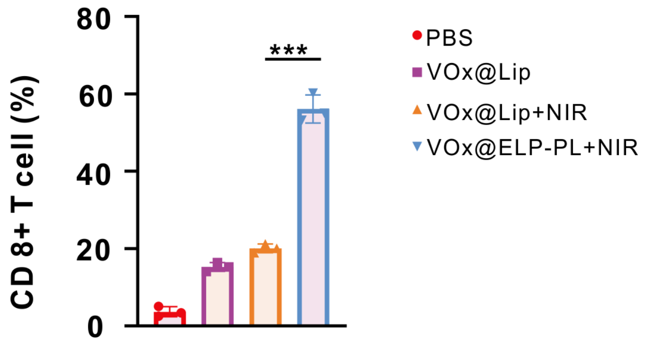

Figure S14. Quantitative flow cytometry analysis of $CD8^+$ T cells after different treatments *in vivo*.

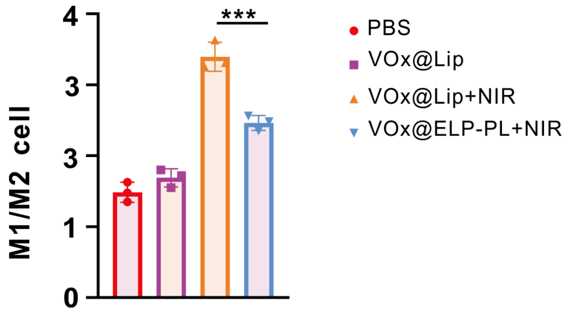

Figure S15. Quantitative flow cytometry analysis of macrophages after different treatments *in vivo*. M1/M2=(Q1+Q2)/(Q2+Q3).

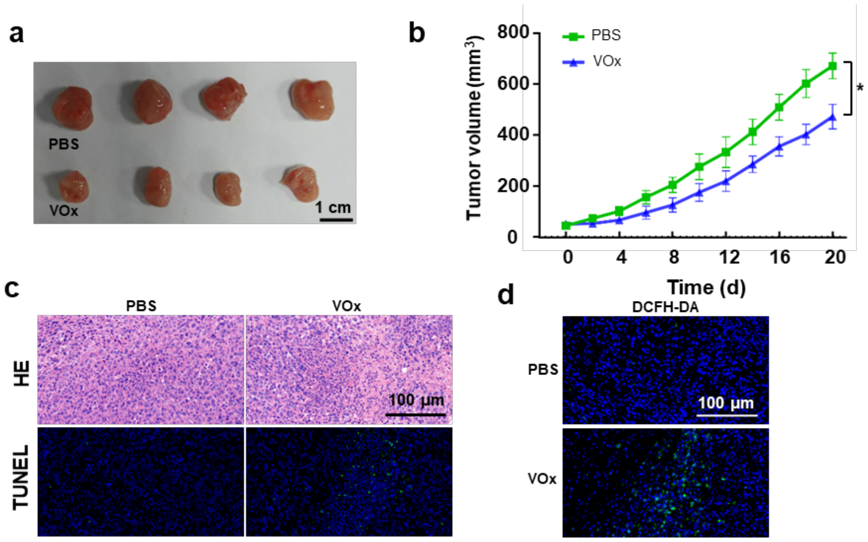

Figure S16. Anti-tumor efficacy of PBS and VO_x in mice bearing subcutaneous CT26 tumors. **a, b.** bright-field image and tumor volume of mice after intratumoral treatment with PBS and VO_x. The data are presented as mean ± standard deviation (n = 4 independent samples). **c, d.** HE, TUNEL, and DCFH-DA staining of tumor sites in subcutaneous CT26 tumor-bearing mice after intratumoral treatment with PBS and VO_x.
